# Supplementary material for: Maternal Lineages of Gepids from Transylvania
Source: Genes (Basel). 2022 Mar 23;13(4):563. doi: 10.3390/genes13040563 (PMC9032604; doi:10.3390/genes13040563)
Supplement: Supplementary file 1 [file genes-13-00563-s001.zip › Maternal Lineages of Gepids from Transylvania_Figure S1.pdf]

Figure S1 - Median Joining Networks for mtDNA sequences of the Gepid group

Phylogenetic trees made with Median Joining Network, from mtDNA sequences of the 46 archaic samples.

Phylogenetic trees are arranged in alphabetic order according to haplogroups. Samples falling into the same sub haplogroup with the studied sample are encircled. The smallest colored circles represent one individual. Circle sizes are proportional to the number of individuals with identical sequences. Green circles identify studied samples, red circles represent modern samples, and violet circles correspond to ancient samples.

Genebank accesion number and origin of samples are listed.

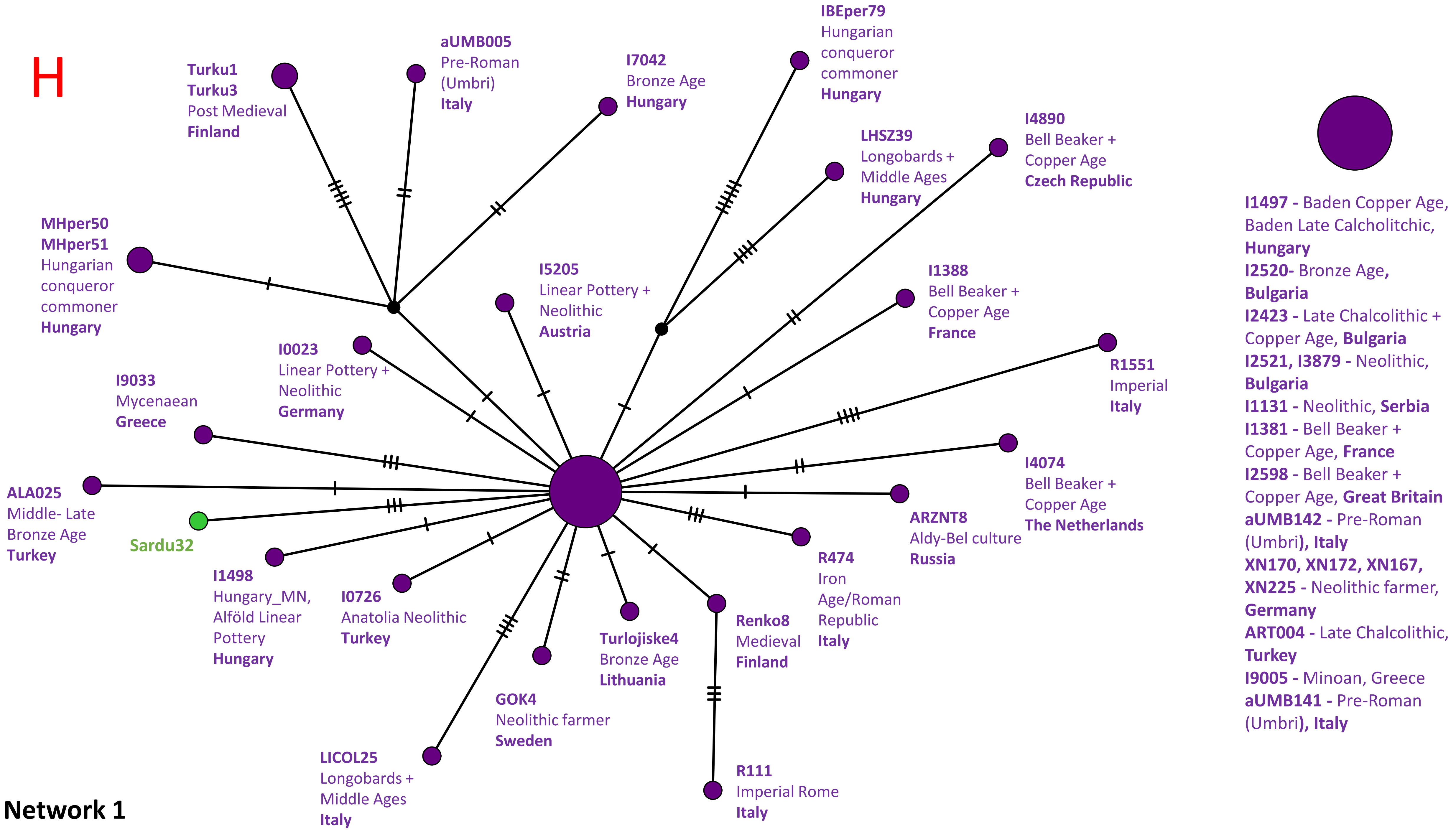

H
Turku1
Turku3
Post Medieval
Finland
aUMB005
Pre-Roman
(Umbri)
Italy
I7042
Bronze Age
Hungary
IBEper79
Hungarian
conqueror
commoner
Hungary
LHSZ39
Longobards +
Middle Ages
Hungary
I4890
Bell Beaker +
Copper Age
Czech Republic
MHper50
MHper51
Hungarian
conqueror
commoner
Hungary
I5205
Linear Pottery +
Neolithic
Austria
I1388
Bell Beaker +
Copper Age
France
I0023
Linear Pottery +
Neolithic
Germany
R1551
Imperial
Italy
I9033
Mycenaean
Greece
I4074
Bell Beaker +
Copper Age
The Netherlands
ALA025
Middle- Late
Bronze Age
Turkey
Sardu32
ARZNT8
Aldy-Bel culture
Russia
I1498
Hungary_MN,
Alföld Linear
Pottery
Hungary
I0726
Anatolia Neolithic
Turkey
R474
Iron
Age/Roman
Republic
Italy
Renko8
Medieval
Finland
Turlojiske4
Bronze Age
Lithuania
GOK4
Neolithic farmer
Sweden
LICOL25
Longobards +
Middle Ages
Italy
R111
Imperial Rome
Italy
I1497 - Baden Copper Age, Baden Late Calcholitchic, Hungary
I2520- Bronze Age, Bulgaria
I2423 - Late Chalcolithic + Copper Age, Bulgaria
I2521, I3879 - Neolithic, Bulgaria
I1131 - Neolithic, Serbia
I1381 - Bell Beaker + Copper Age, France
I2598 - Bell Beaker + Copper Age, Great Britain
aUMB142 - Pre-Roman (Umbri), Italy
XN170, XN172, XN167, XN225 - Neolithic farmer, Germany
ART004 - Late Chalcolithic, Turkey
I9005 - Minoan, Greece
aUMB141 - Pre-Roman (Umbri), Italy

Network 1

H1

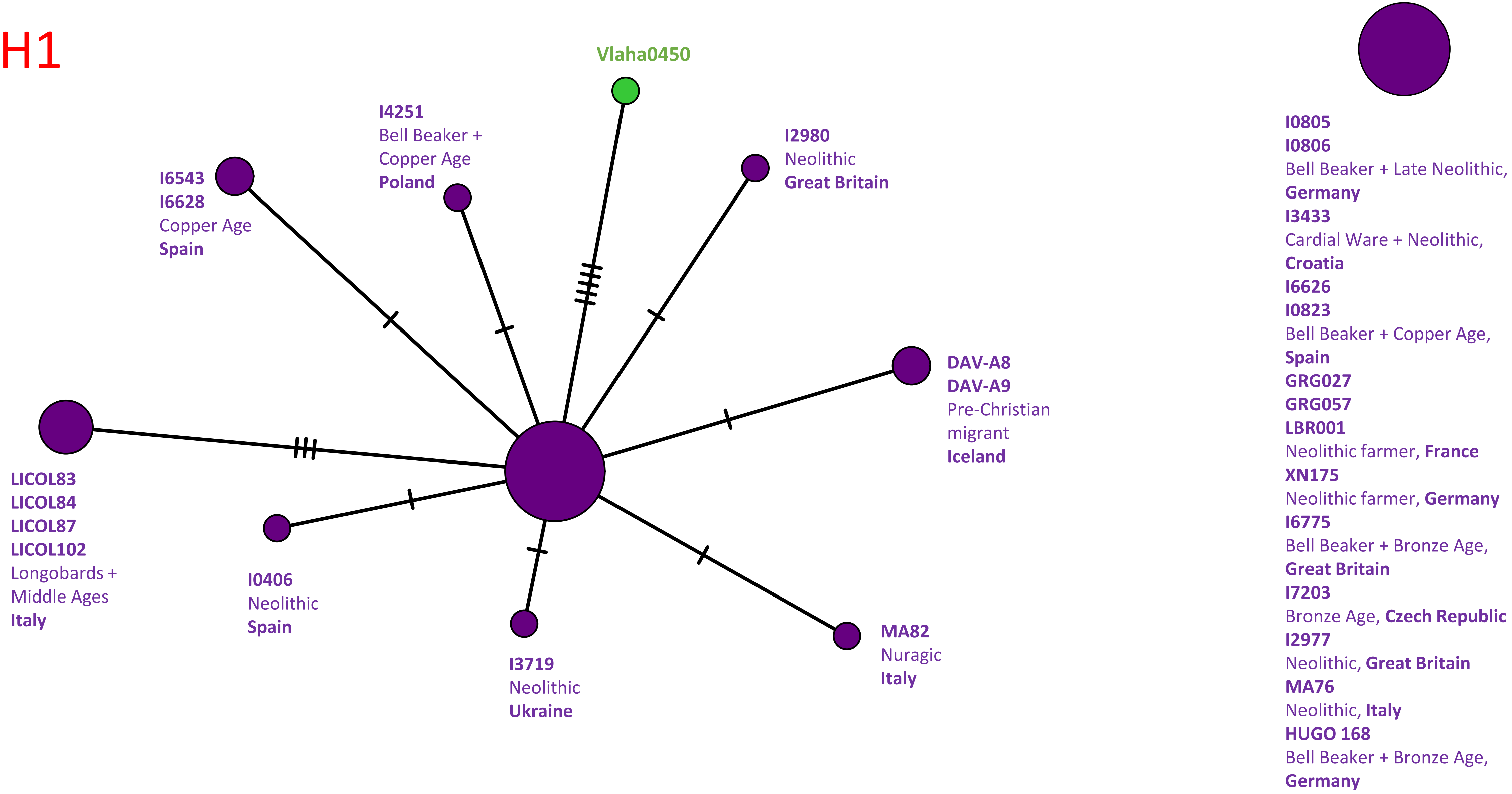
Vlaha0450
I4251
Bell Beaker +
Copper Age
Poland
I6543
I6628
Copper Age
Spain
I2980
Neolithic
Great Britain
DAV-A8
DAV-A9
Pre-Christian
migrant
Iceland
LICOL83
LICOL84
LICOL87
LICOL102
Longobards +
Middle Ages
Italy
I0406
Neolithic
Spain
I3719
Neolithic
Ukraine
MA82
Nuragic
Italy
I0805
I0806
Bell Beaker + Late Neolithic,
Germany
I3433
Cardial Ware + Neolithic,
Croatia
I6626
I0823
Bell Beaker + Copper Age,
Spain
GRG027
GRG057
LBR001
Neolithic farmer, France
XN175
Neolithic farmer, Germany
I6775
Bell Beaker + Bronze Age,
Great Britain
I7203
Bronze Age, Czech Republic
I2977
Neolithic, Great Britain
MA76
Neolithic, Italy
HUGO 168
Bell Beaker + Bronze Age,
Germany

Network 2

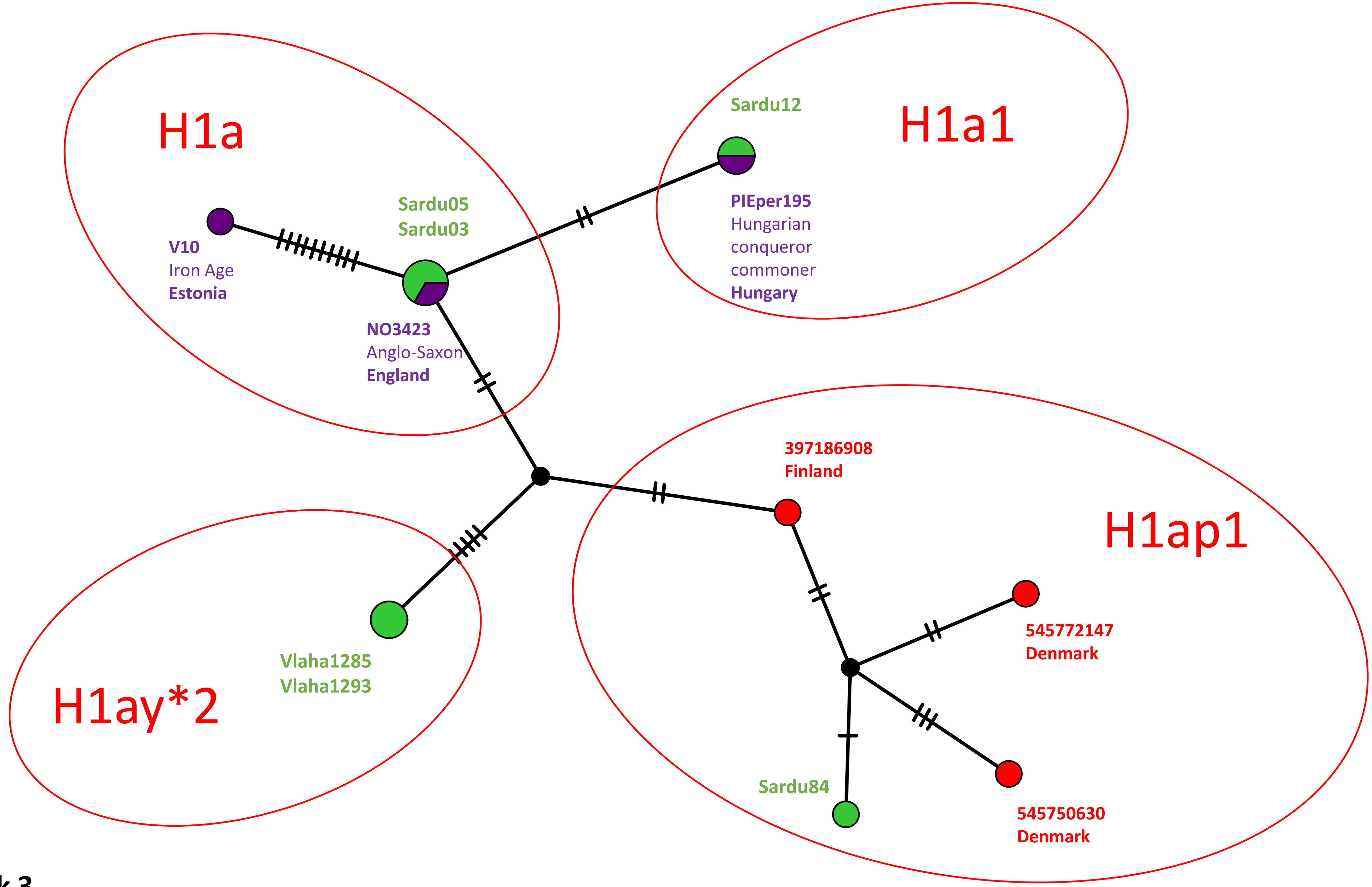
H1a
V10
Iron Age
Estonia
Sardu05
Sardu03
NO3423
Anglo-Saxon
England
Sardu12
H1a1
PIEper195
Hungarian
conqueror
commoner
Hungary
397186908
Finland
H1ap1
545772147
Denmark
545750630
Denmark
Sardu84
Vlaha1285
Vlaha1293
H1ay*2

Network 3

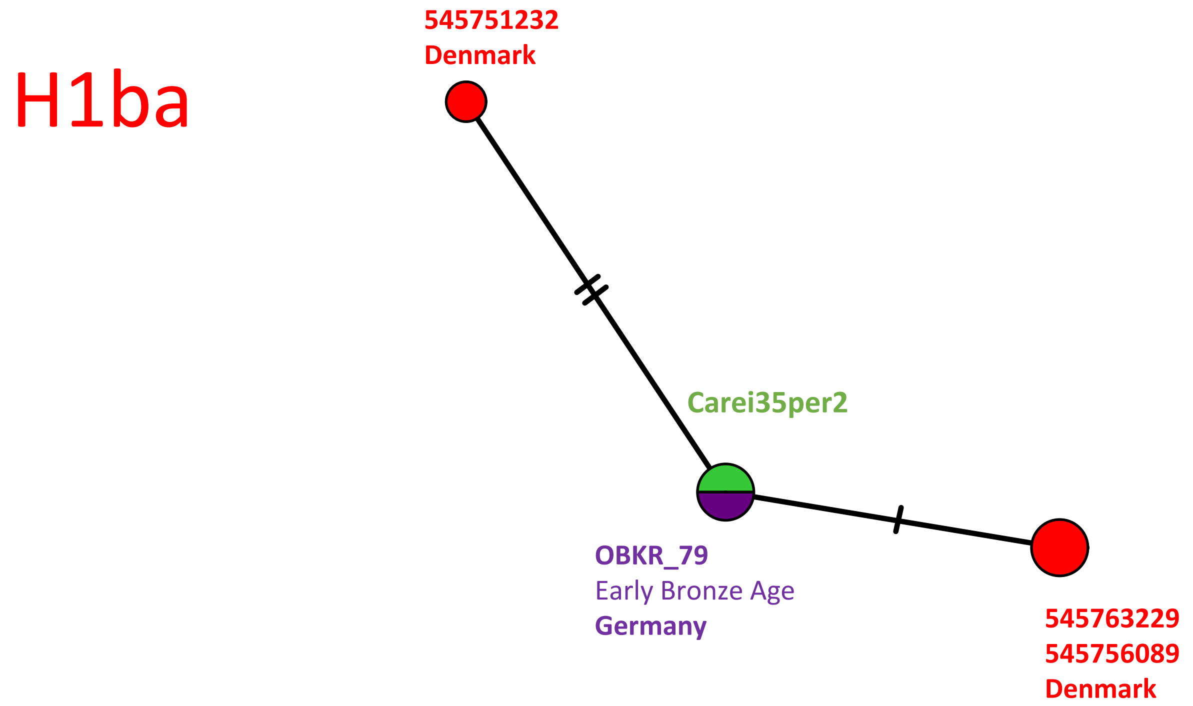

H1ba
545751232
Denmark
Carei35per2
OBKR_79
Early Bronze Age
Germany
545763229
545756089
Denmark

Network 4

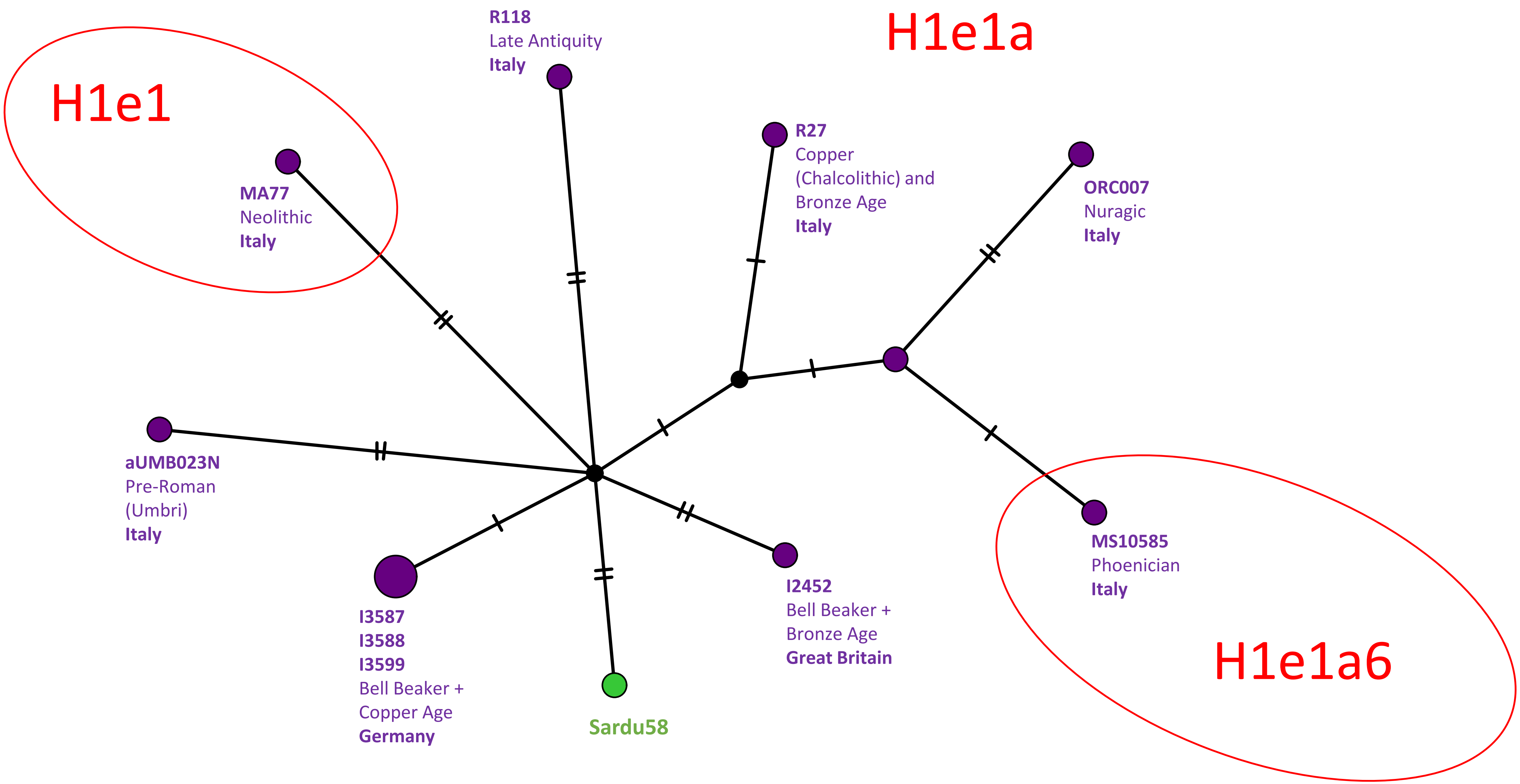

H1e1
H1e1a
H1e1a6
R118
Late Antiquity
Italy
MA77
Neolithic
Italy
R27
Copper
(Chalcolithic) and
Bronze Age
Italy
ORC007
Nuragic
Italy
aUMB023N
Pre-Roman
(Umbri)
Italy
MS10585
Phoenician
Italy
I3587
I3588
I3599
Bell Beaker +
Copper Age
Germany
I2452
Bell Beaker +
Bronze Age
Great Britain
Sardu58

Network 5

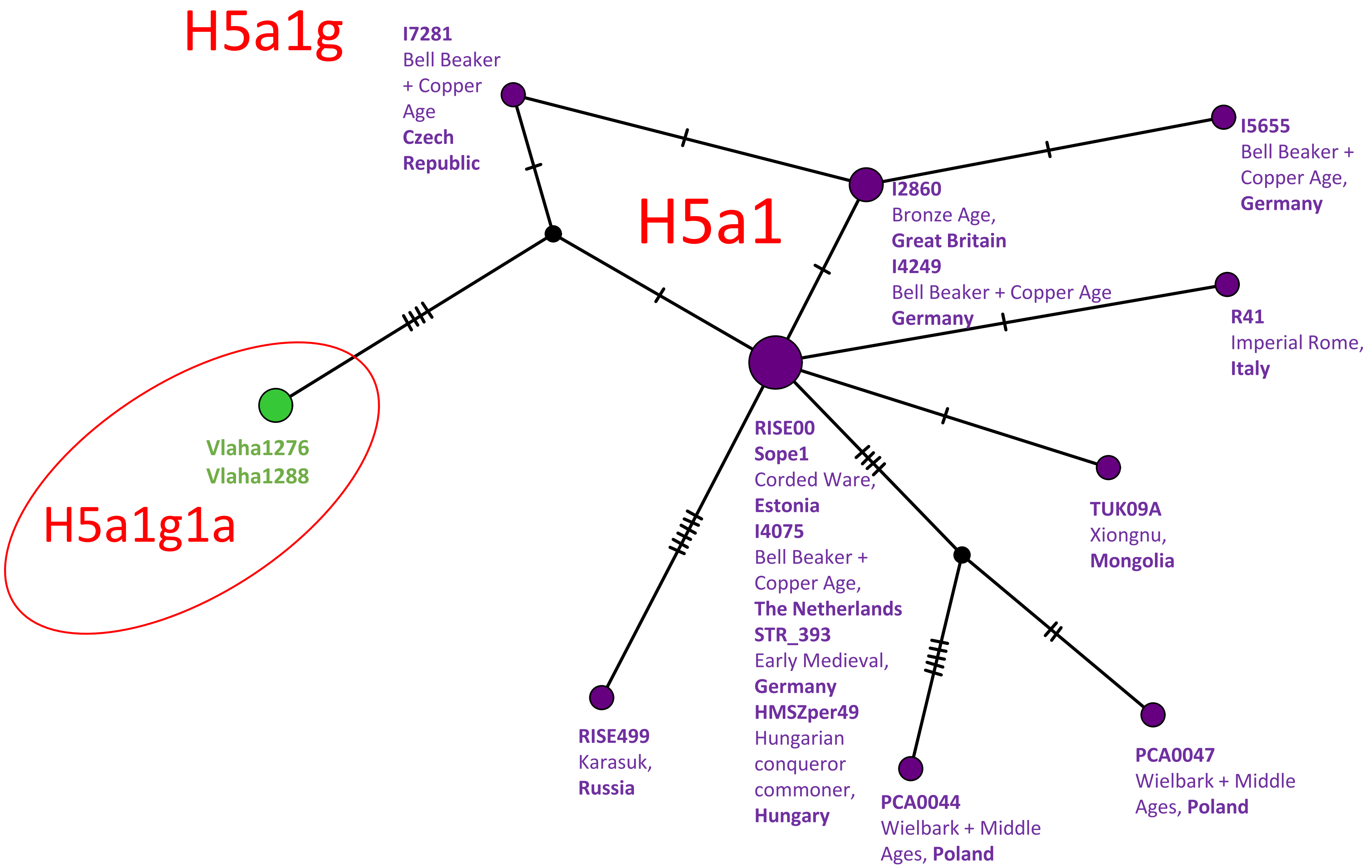
H5a1g
I7281 Bell Beaker + Copper Age Czech Republic
H5a1
I5655 Bell Beaker + Copper Age, Germany
I2860 Bronze Age, Great Britain
I4249 Bell Beaker + Copper Age Germany
R41 Imperial Rome, Italy
Vlaha1276
Vlaha1288
H5a1g1a
RISE00
Sope1
Corded Ware, Estonia
I4075 Bell Beaker + Copper Age, The Netherlands
STR_393 Early Medieval, Germany
HMSZper49 Hungarian conqueror commoner, Hungary
TUK09A Xiongnu, Mongolia
RISE499 Karasuk, Russia
PCA0044 Wielbark + Middle Ages, Poland
PCA0047 Wielbark + Middle Ages, Poland

Network 6

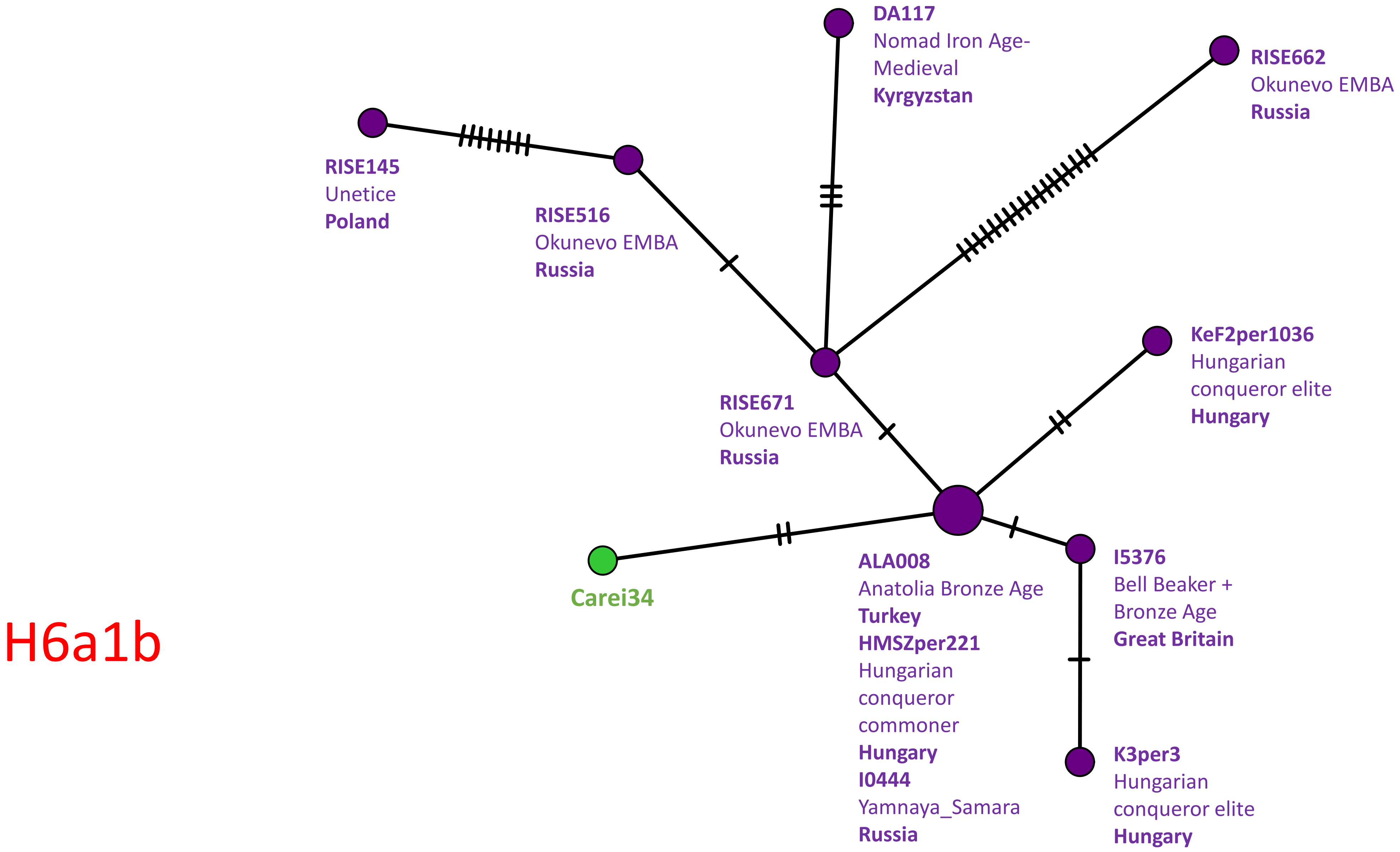
DA117
Nomad Iron Age-
Medieval
Kyrgyzstan
RISE662
Okunevo EMBA
Russia
RISE145
Unetice
Poland
RISE516
Okunevo EMBA
Russia
KeF2per1036
Hungarian
conqueror elite
Hungary
RISE671
Okunevo EMBA
Russia
Carei34
ALA008
Anatolia Bronze Age
Turkey
HMSZper221
Hungarian
conqueror
commoner
Hungary
I0444
Yamnaya_Samara
Russia
I5376
Bell Beaker +
Bronze Age
Great Britain
K3per3
Hungarian
conqueror elite
Hungary
H6a1b

Network 7

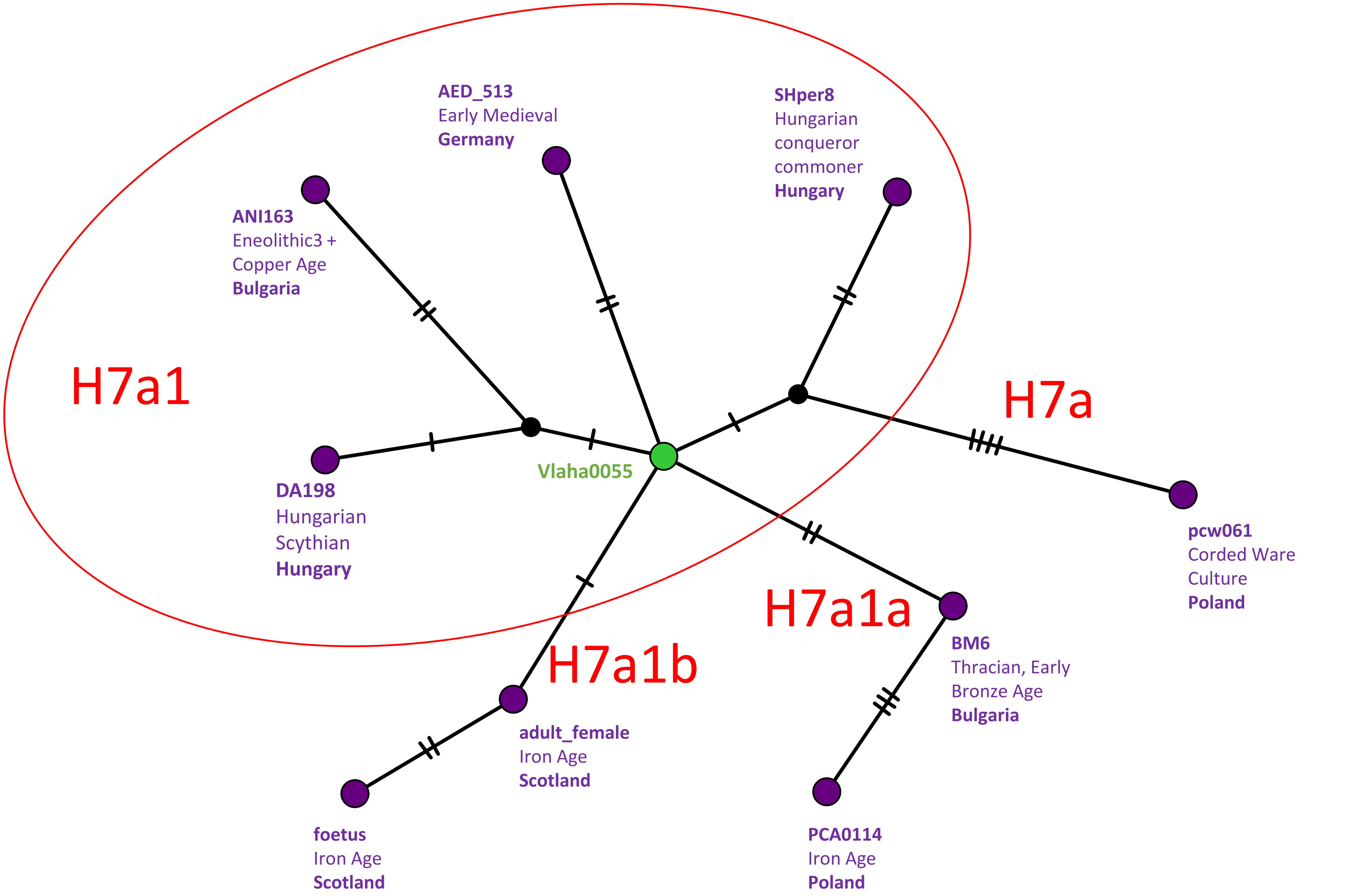

AED_513
Early Medieval
Germany
SHper8
Hungarian
conqueror
commoner
Hungary
ANI163
Eneolithic3 +
Copper Age
Bulgaria
H7a1
H7a
DA198
Hungarian
Scythian
Hungary
Vlaha0055
pcw061
Corded Ware
Culture
Poland
H7a1a
BM6
Thracian, Early
Bronze Age
Bulgaria
H7a1b
adult_female
Iron Age
Scotland
foetus
Iron Age
Scotland
PCA0114
Iron Age
Poland

Network 8

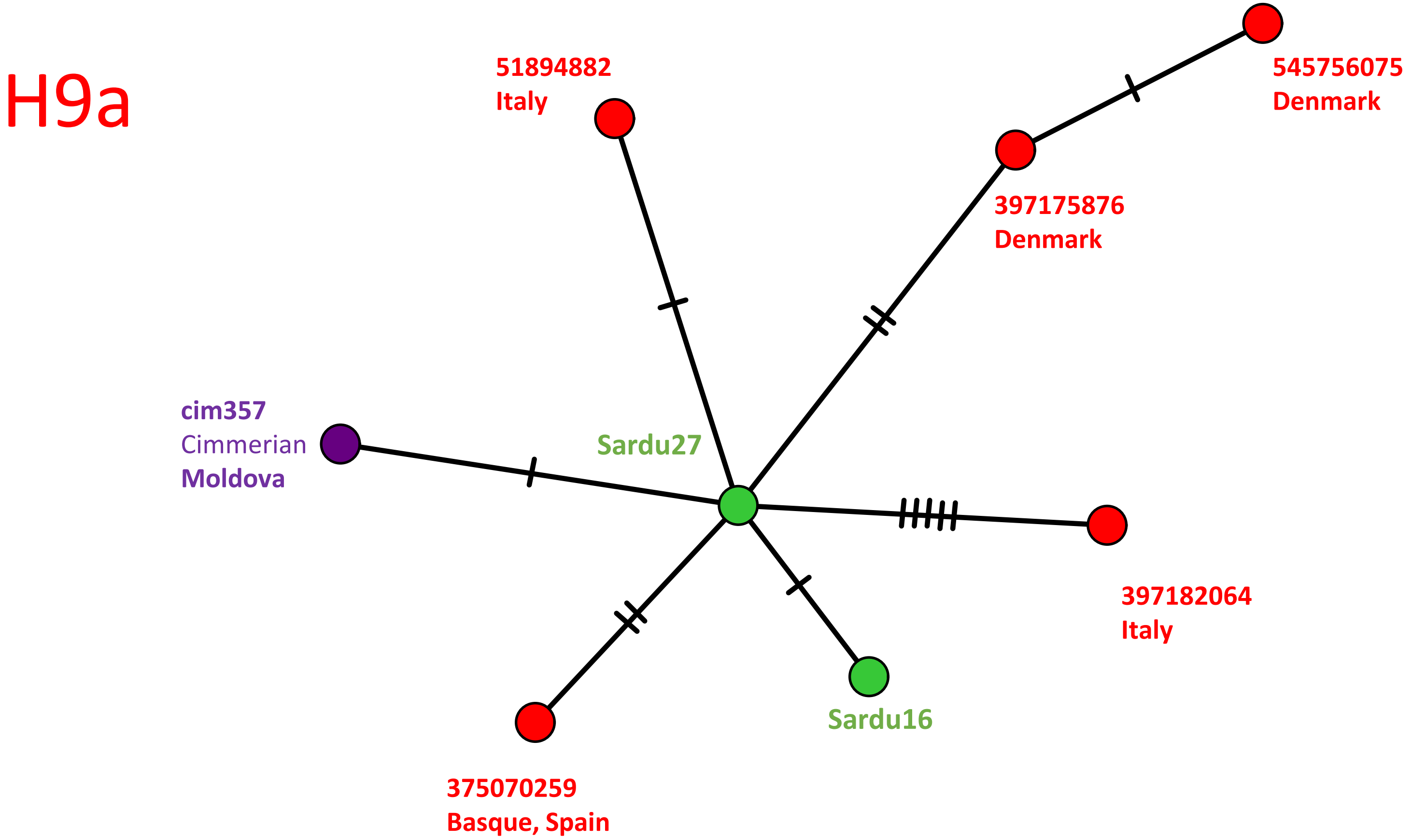
H9a
51894882
Italy
545756075
Denmark
397175876
Denmark
cim357
Cimmerian
Moldova
Sardu27
397182064
Italy
Sardu16
375070259
Basque, Spain

Network 9

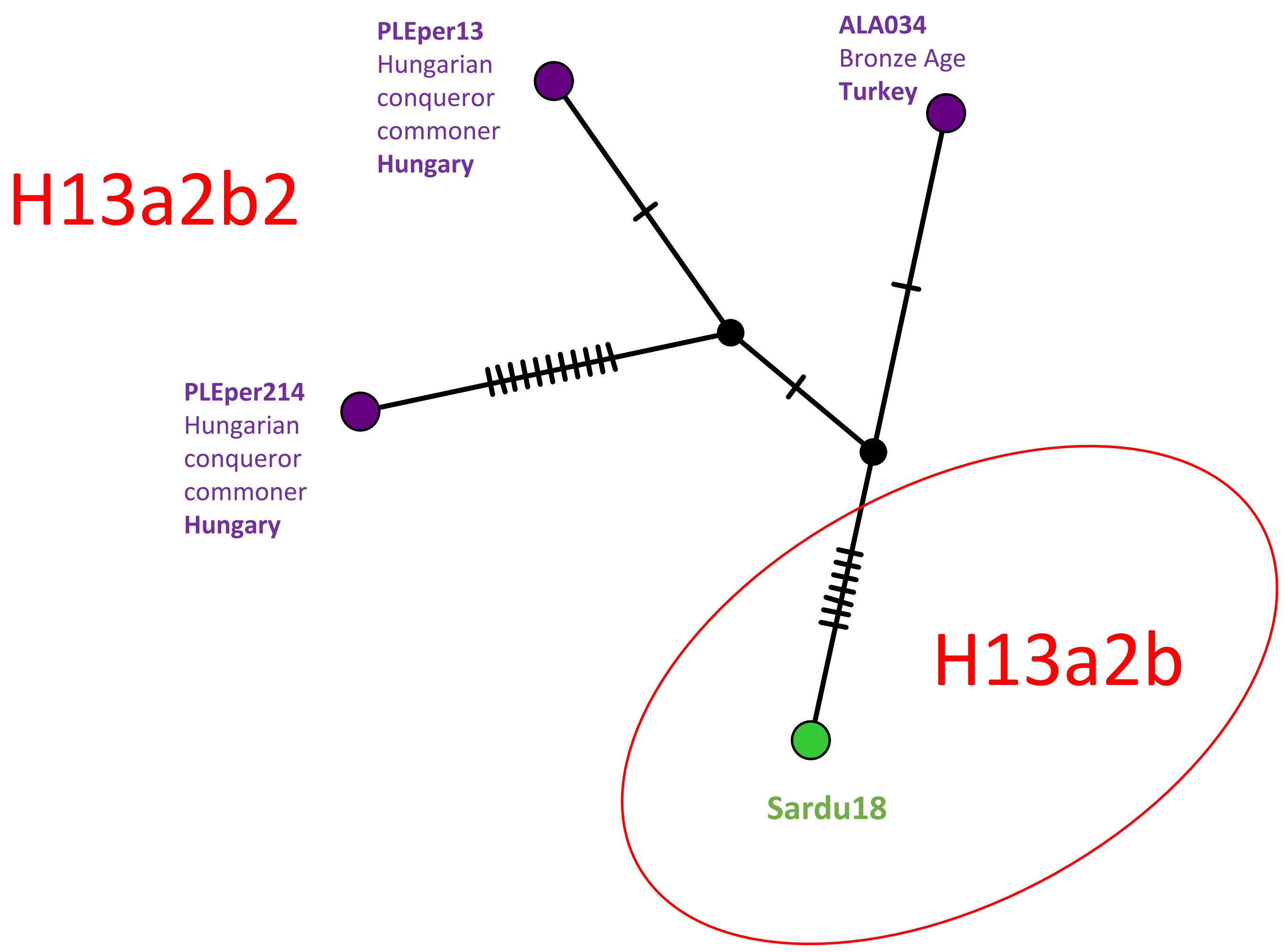
PLEper13
Hungarian
conqueror
commoner
Hungary
ALA034
Bronze Age
Turkey
H13a2b2
PLEper214
Hungarian
conqueror
commoner
Hungary
H13a2b
Sardu18

Network 10

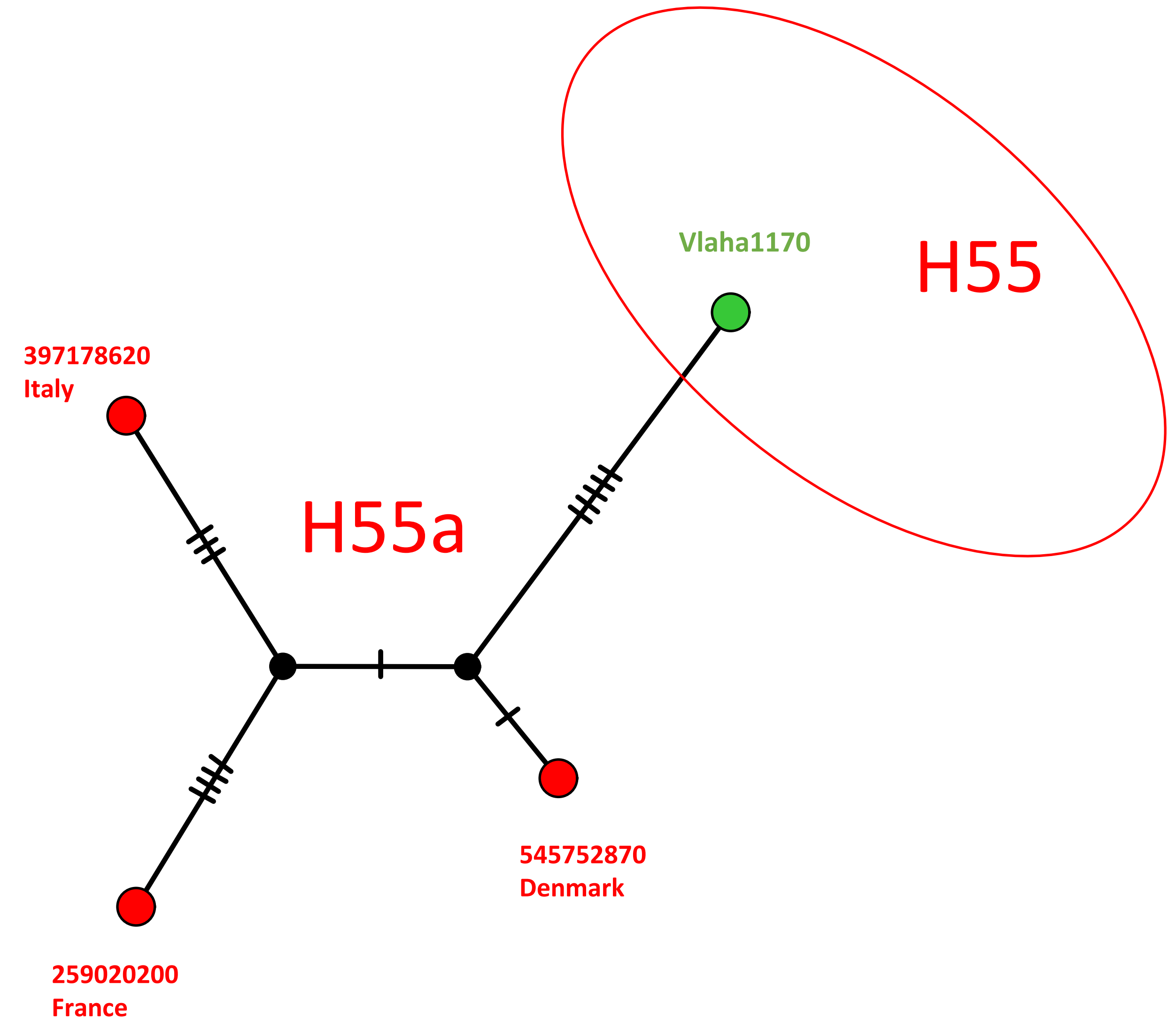
Vlaha1170
H55
397178620
Italy
H55a
545752870
Denmark
259020200
France

Network 11

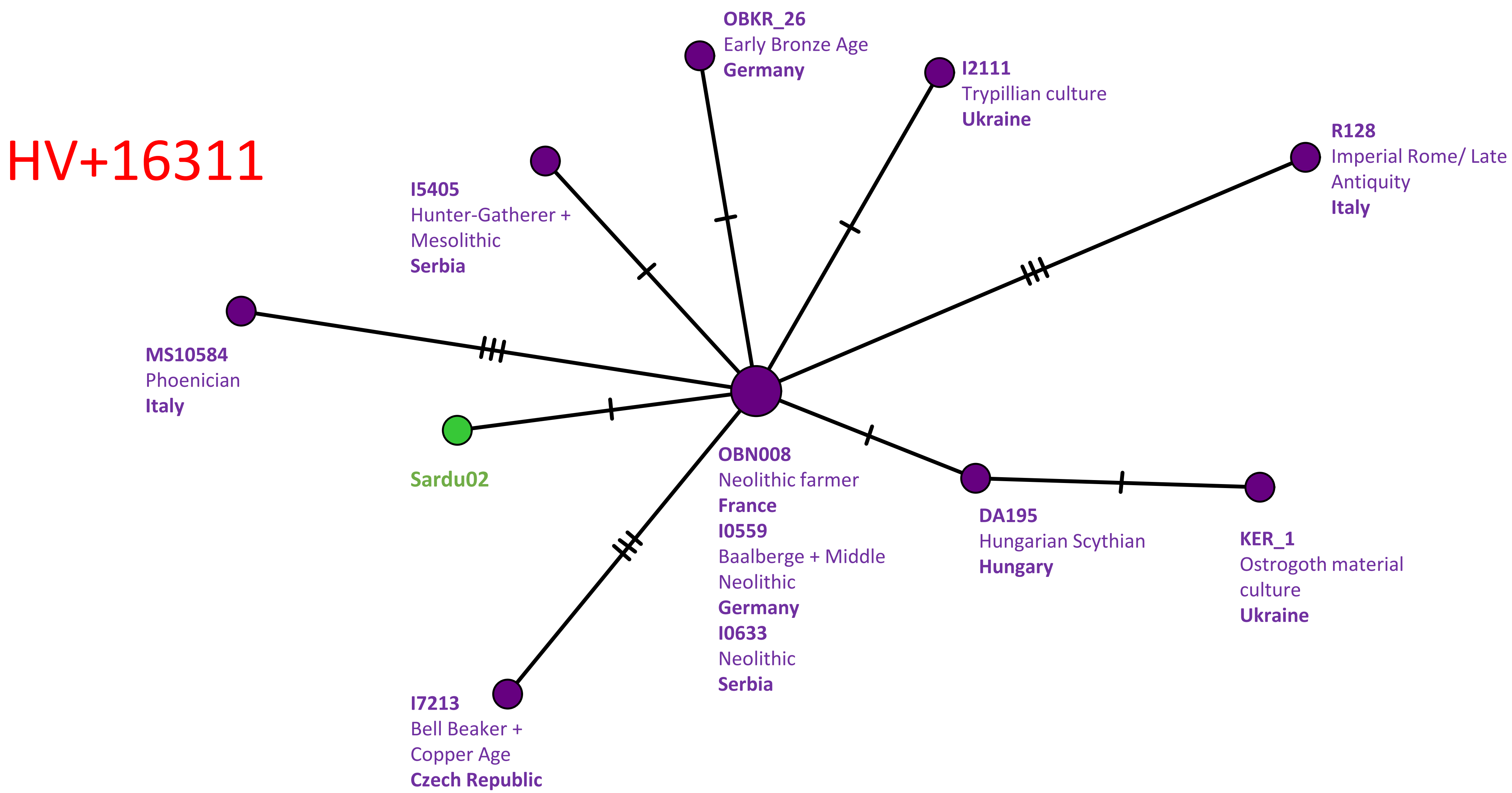

HV+16311
OBKR_26
Early Bronze Age
Germany
I2111
Trypillian culture
Ukraine
R128
Imperial Rome/ Late Antiquity
Italy
I5405
Hunter-Gatherer + Mesolithic
Serbia
MS10584
Phoenician
Italy
Sardu02
OBN008
Neolithic farmer
France
I0559
Baalberge + Middle Neolithic
Germany
I0633
Neolithic
Serbia
DA195
Hungarian Scythian
Hungary
KER_1
Ostrogoth material culture
Ukraine
I7213
Bell Beaker + Copper Age
Czech Republic

Network 12

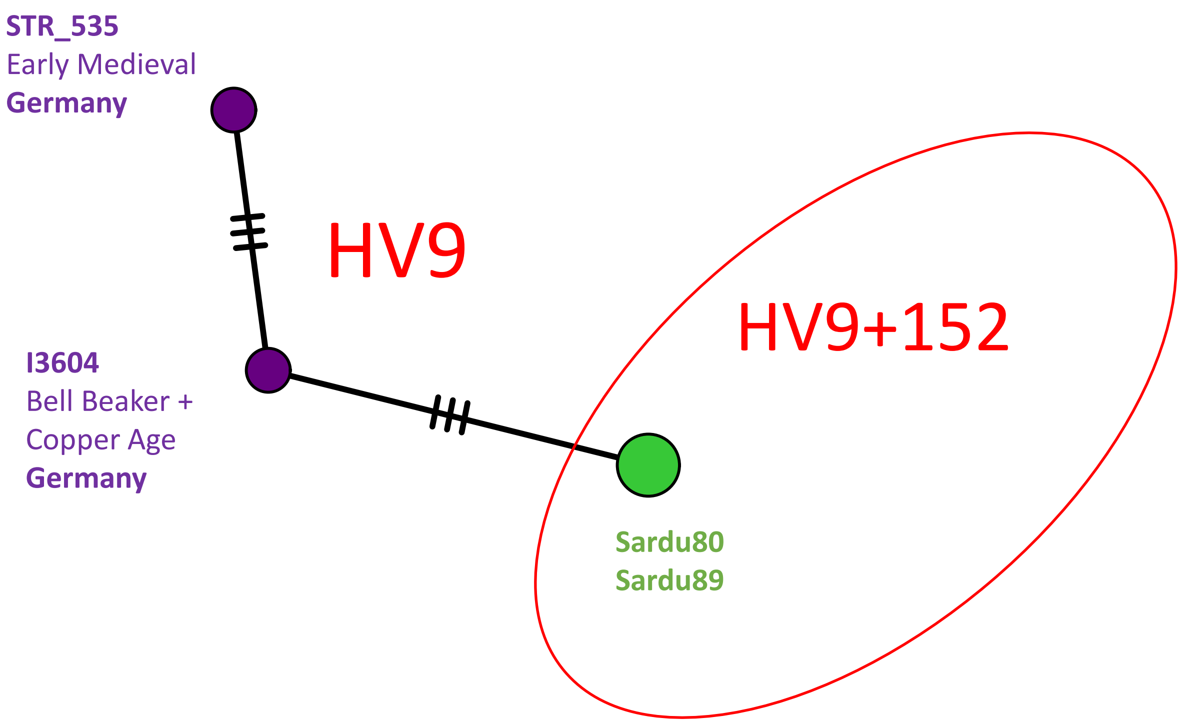
STR_535
Early Medieval
Germany
HV9
I3604
Bell Beaker +
Copper Age
Germany
HV9+152
Sardu80
Sardu89

Network 13

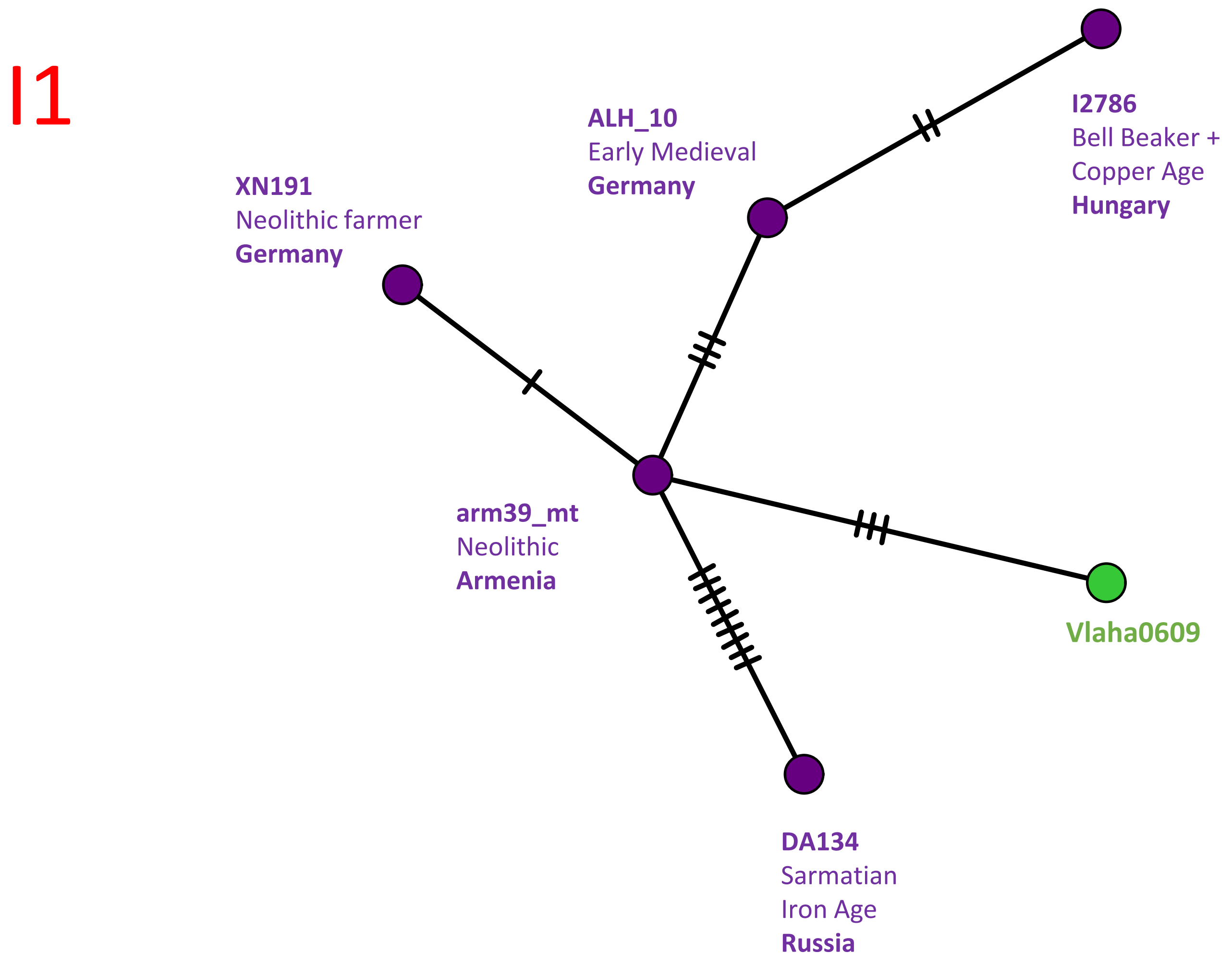
I1
XN191
Neolithic farmer
Germany
ALH_10
Early Medieval
Germany
I2786
Bell Beaker +
Copper Age
Hungary
arm39_mt
Neolithic
Armenia
Vlaha0609
DA134
Sarmatian
Iron Age
Russia

Network 14

I1a1a3

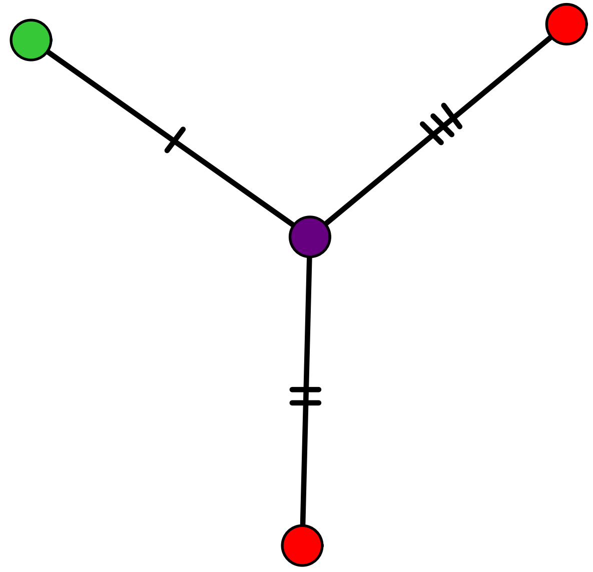

Network 15

I1b

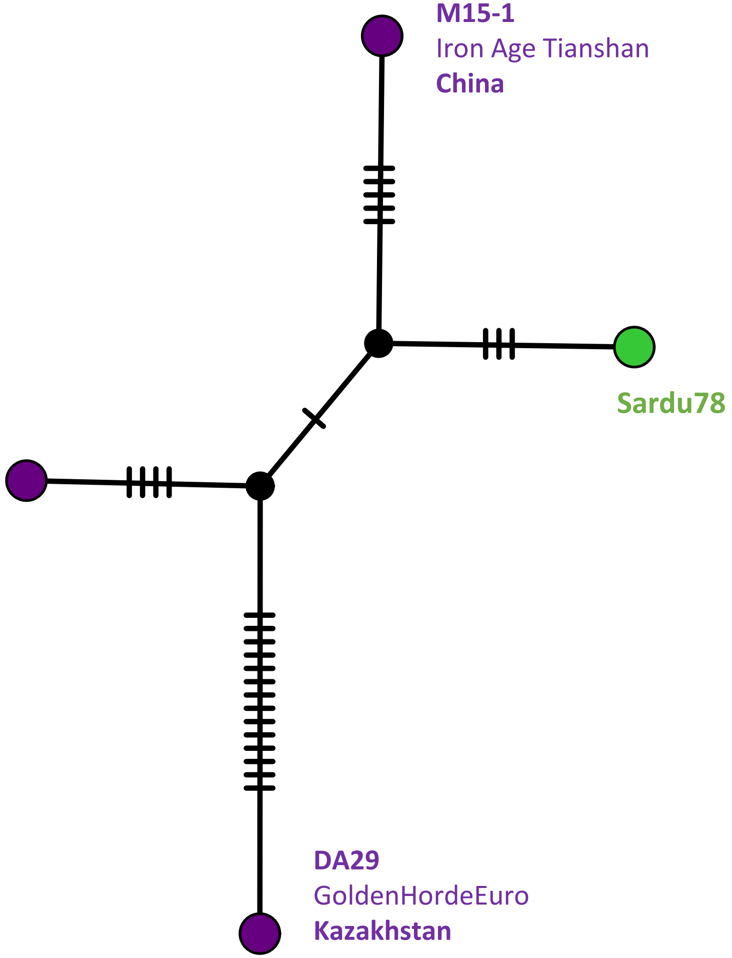

Network 16

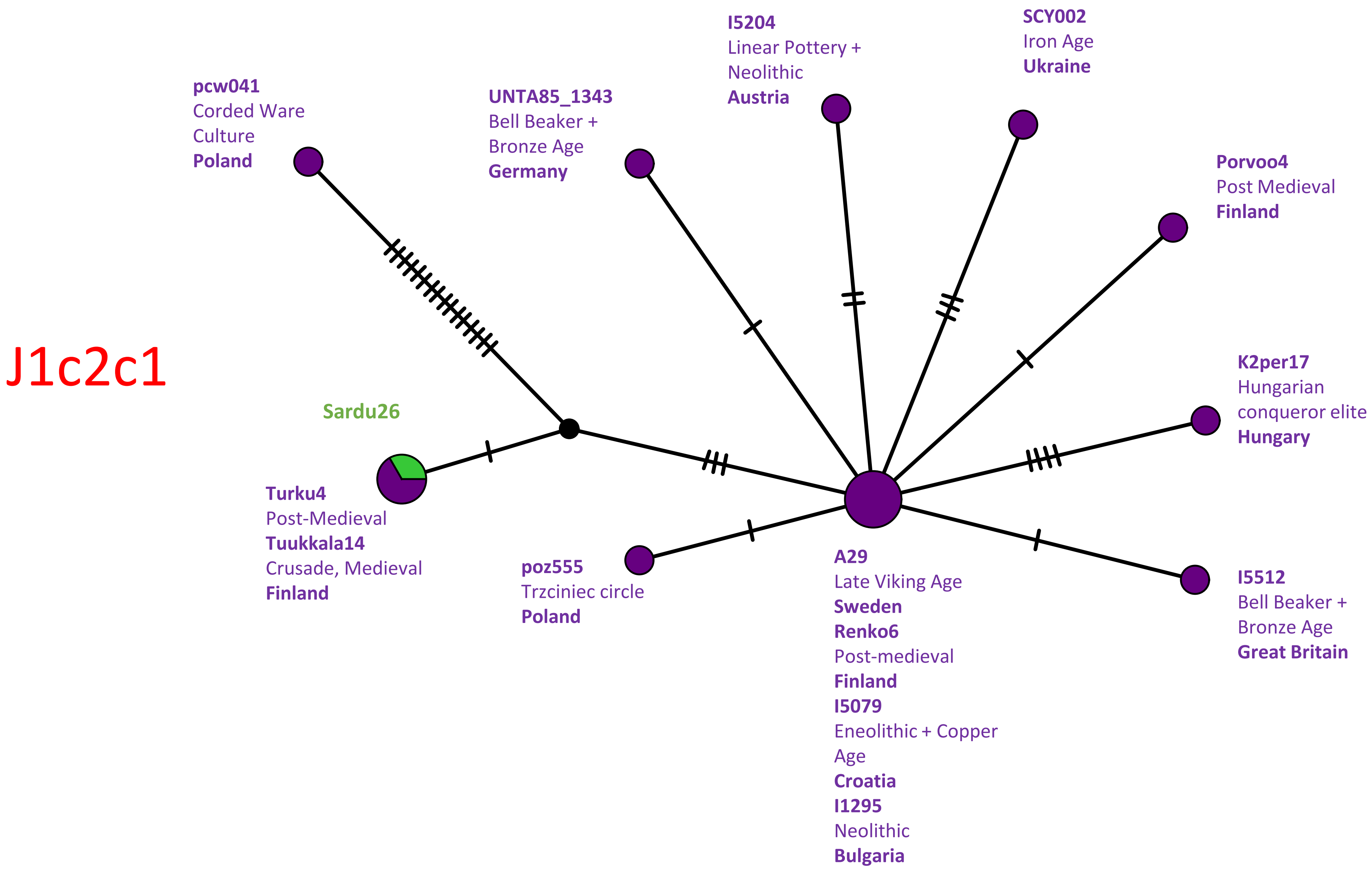
J1c2c1
pcw041
Corded Ware
Culture
Poland
UNTA85_1343
Bell Beaker +
Bronze Age
Germany
I5204
Linear Pottery +
Neolithic
Austria
SCY002
Iron Age
Ukraine
Porvoo4
Post Medieval
Finland
K2per17
Hungarian
conqueror elite
Hungary
Sardu26
Turku4
Post-Medieval
Tuukkala14
Crusade, Medieval
Finland
poz555
Trzciniec circle
Poland
A29
Late Viking Age
Sweden
Renko6
Post-medieval
Finland
I5079
Eneolithic + Copper
Age
Croatia
I1295
Neolithic
Bulgaria
I5512
Bell Beaker +
Bronze Age
Great Britain

Network 17

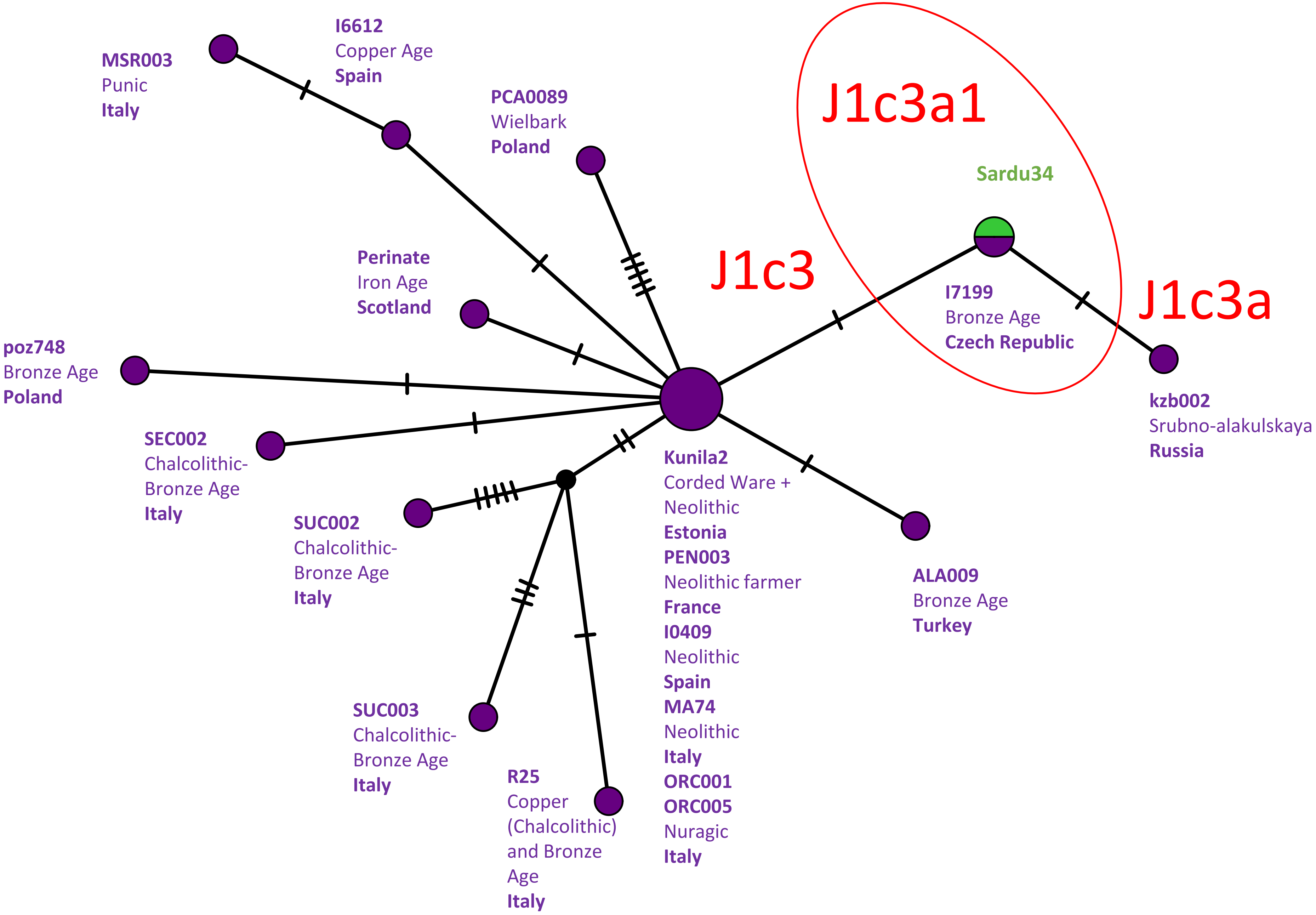
J1c3a1
Sardu34
J1c3
I7199
Bronze Age
Czech Republic
J1c3a
kzb002
Srubno-alakulskaya
Russia
I6612
Copper Age
Spain
MSR003
Punic
Italy
PCA0089
Wielbark
Poland
Perinate
Iron Age
Scotland
poz748
Bronze Age
Poland
SEC002
Chalcolithic-
Bronze Age
Italy
SUC002
Chalcolithic-
Bronze Age
Italy
Kunila2
Corded Ware +
Neolithic
Estonia
PEN003
Neolithic farmer
France
I0409
Neolithic
Spain
MA74
Neolithic
Italy
ORC001
ORC005
Nuragic
Italy
ALA009
Bronze Age
Turkey
SUC003
Chalcolithic-
Bronze Age
Italy
R25
Copper
(Chalcolithic)
and Bronze
Age
Italy

Network 18

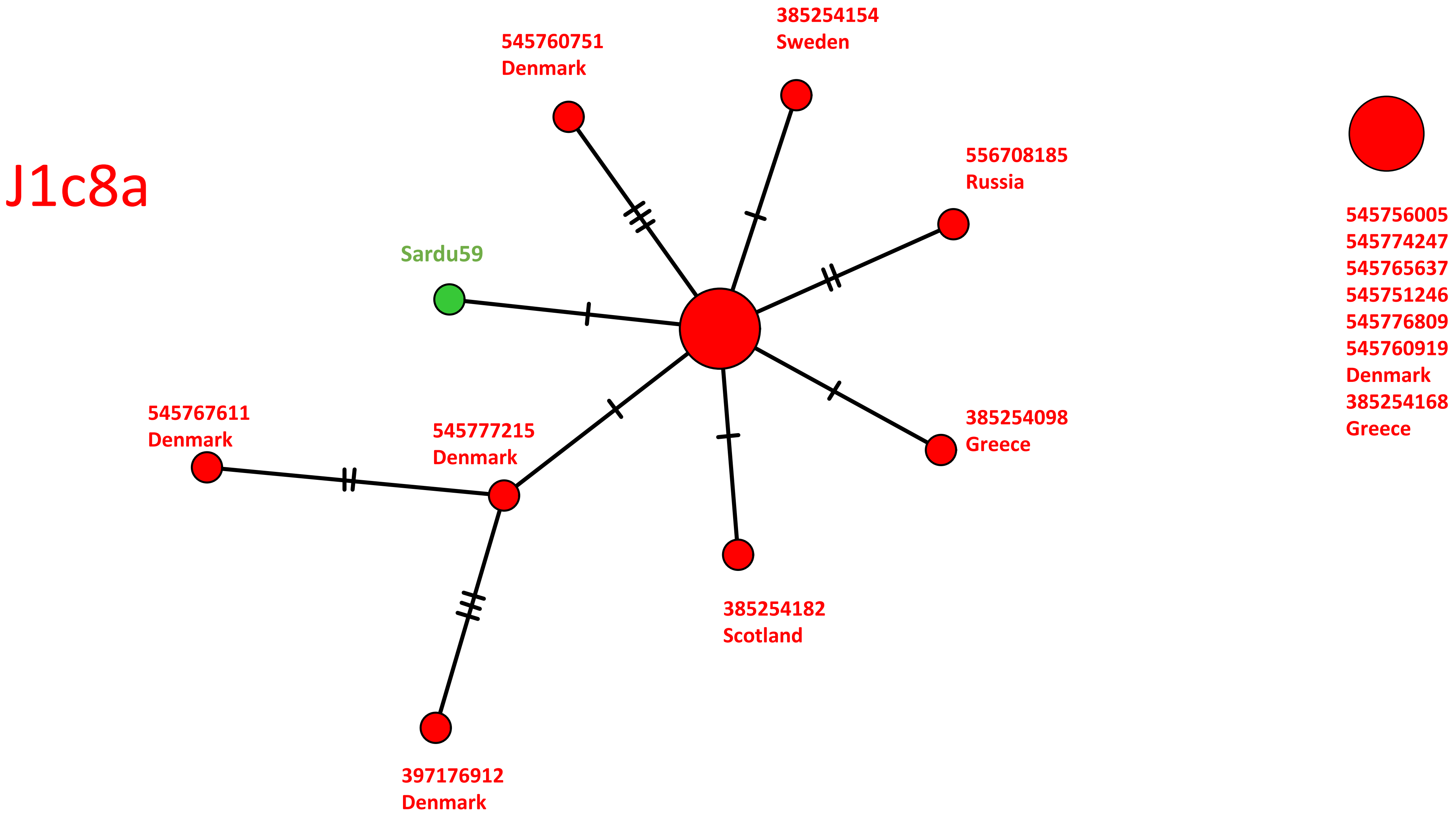
J1c8a
545760751
Denmark
385254154
Sweden
556708185
Russia
Sardu59
545767611
Denmark
545777215
Denmark
385254098
Greece
385254182
Scotland
397176912
Denmark
545756005
545774247
545765637
545751246
545776809
545760919
Denmark
385254168
Greece

Network 19

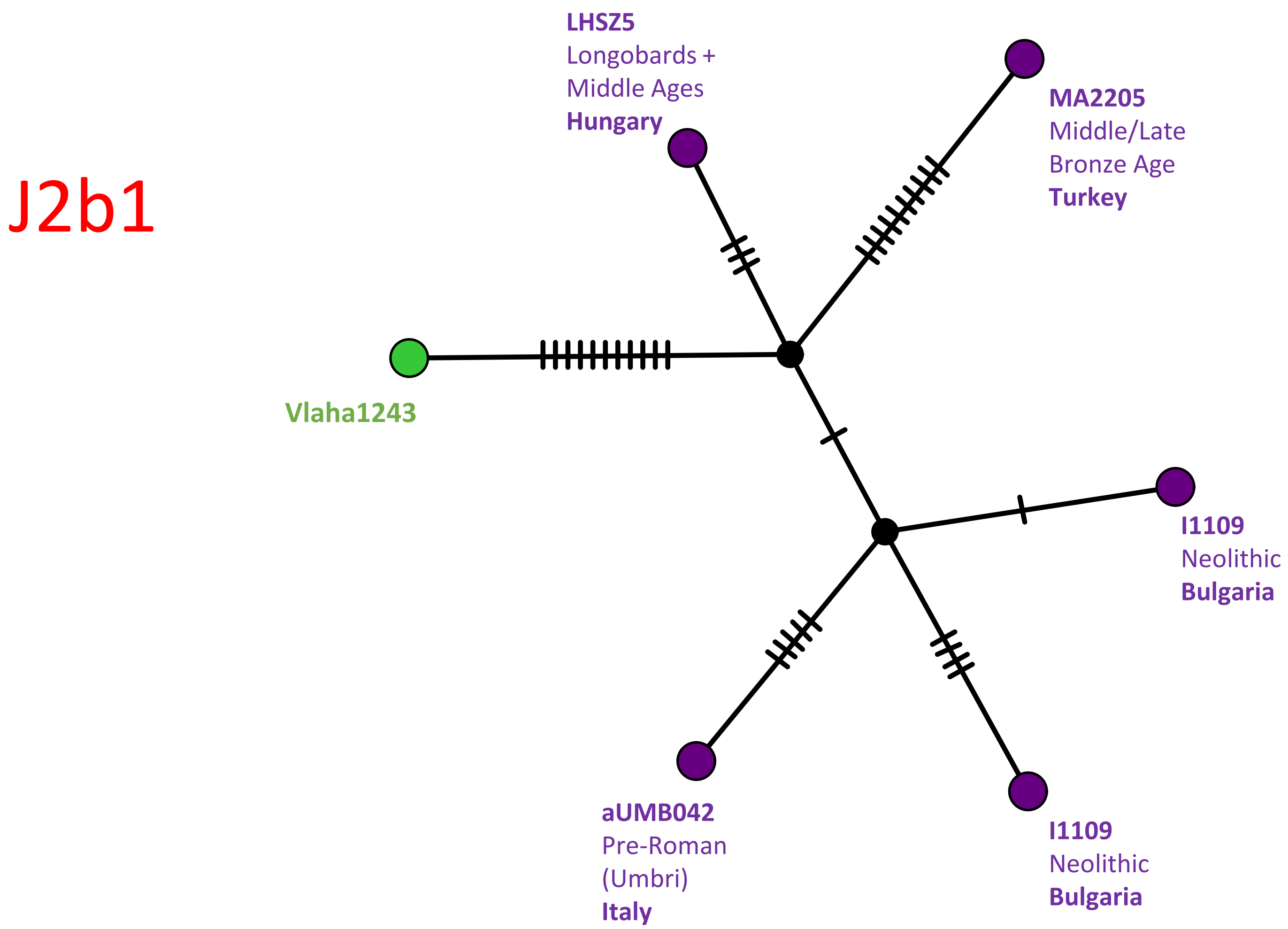
J2b1
LHSZ5
Longobards +
Middle Ages
Hungary
MA2205
Middle/Late
Bronze Age
Turkey
Vlaha1243
I1109
Neolithic
Bulgaria
aUMB042
Pre-Roman
(Umbri)
Italy
I1109
Neolithic
Bulgaria

Network 20

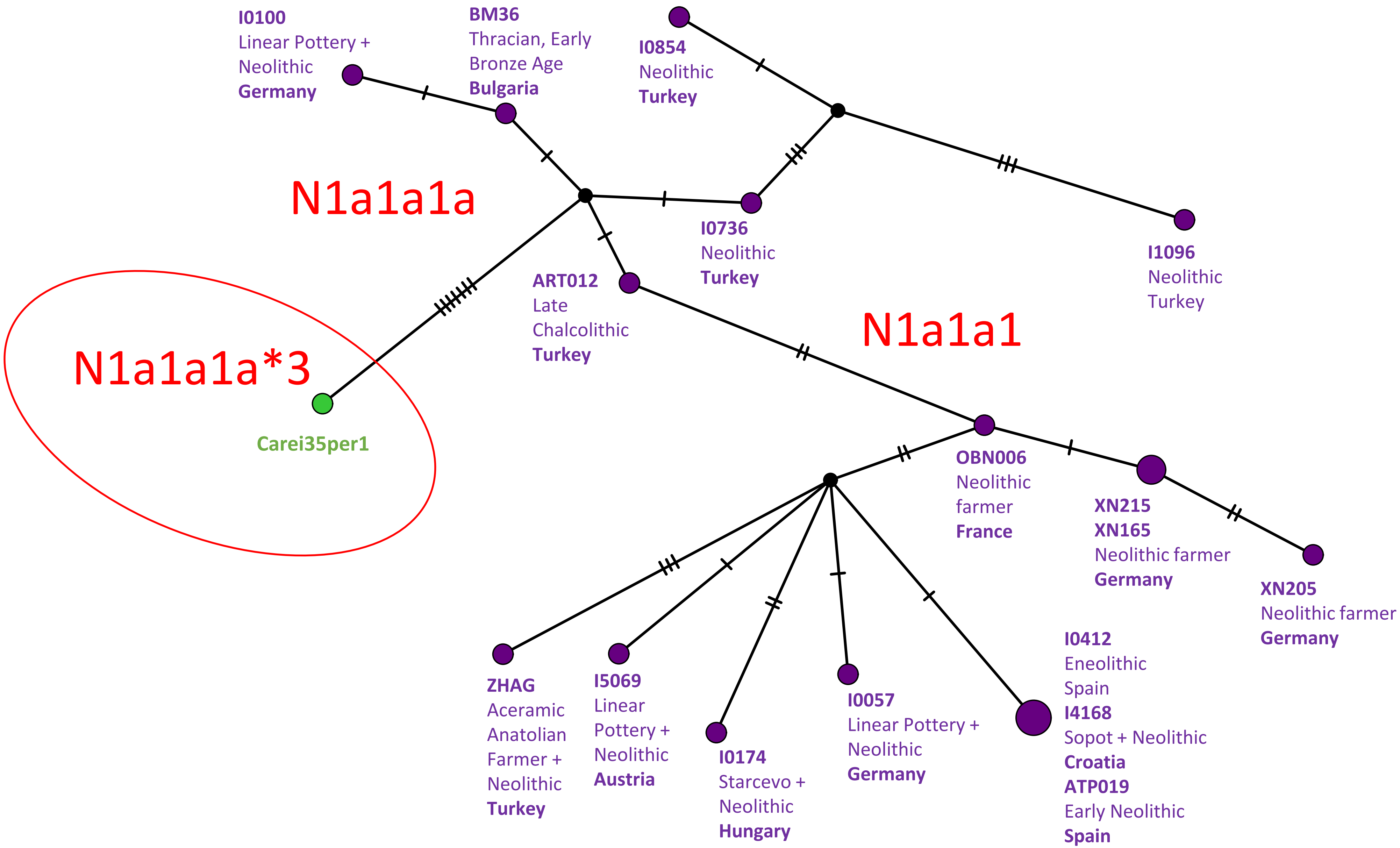
I0100
Linear Pottery +
Neolithic
Germany
BM36
Thracian, Early
Bronze Age
Bulgaria
I0854
Neolithic
Turkey
N1a1a1a
I0736
Neolithic
Turkey
I1096
Neolithic
Turkey
ART012
Late
Chalcolithic
Turkey
N1a1a1
N1a1a1a*3
Carei35per1
OBN006
Neolithic
farmer
France
XN215
XN165
Neolithic farmer
Germany
XN205
Neolithic farmer
Germany
I0412
Eneolithic
Spain
I4168
Sopot + Neolithic
Croatia
ATP019
Early Neolithic
Spain
ZHAG
Aceramic
Anatolian
Farmer +
Neolithic
Turkey
I5069
Linear
Pottery +
Neolithic
Austria
I0174
Starcevo +
Neolithic
Hungary
I0057
Linear Pottery +
Neolithic
Germany

Network 21

T1

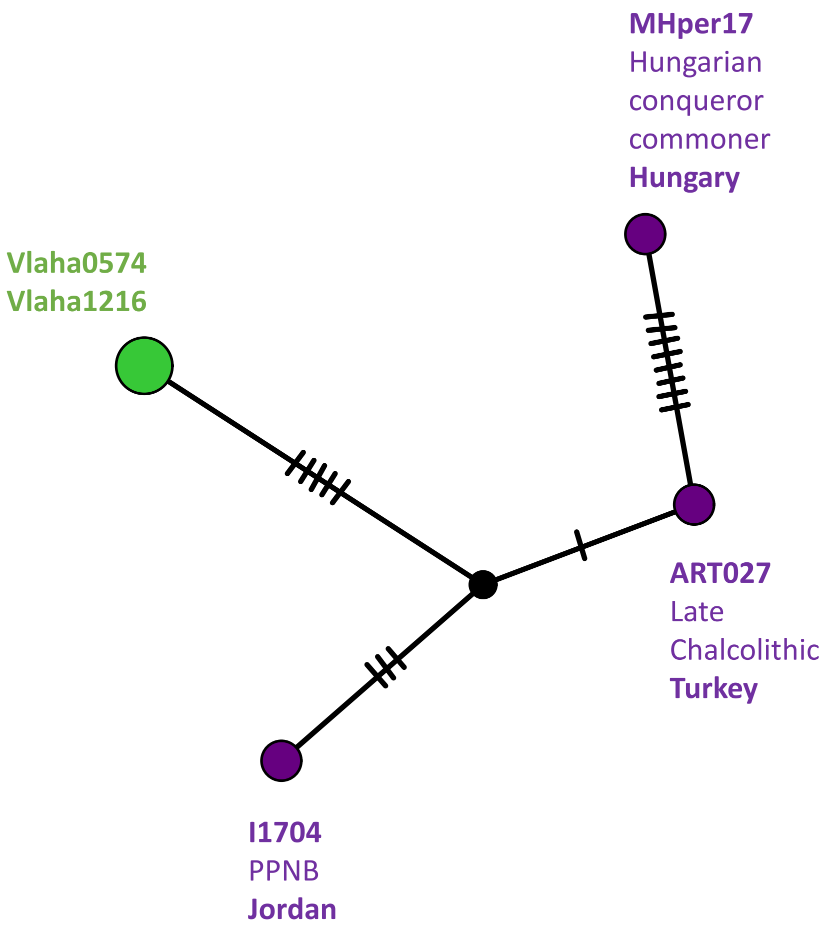

Network 22

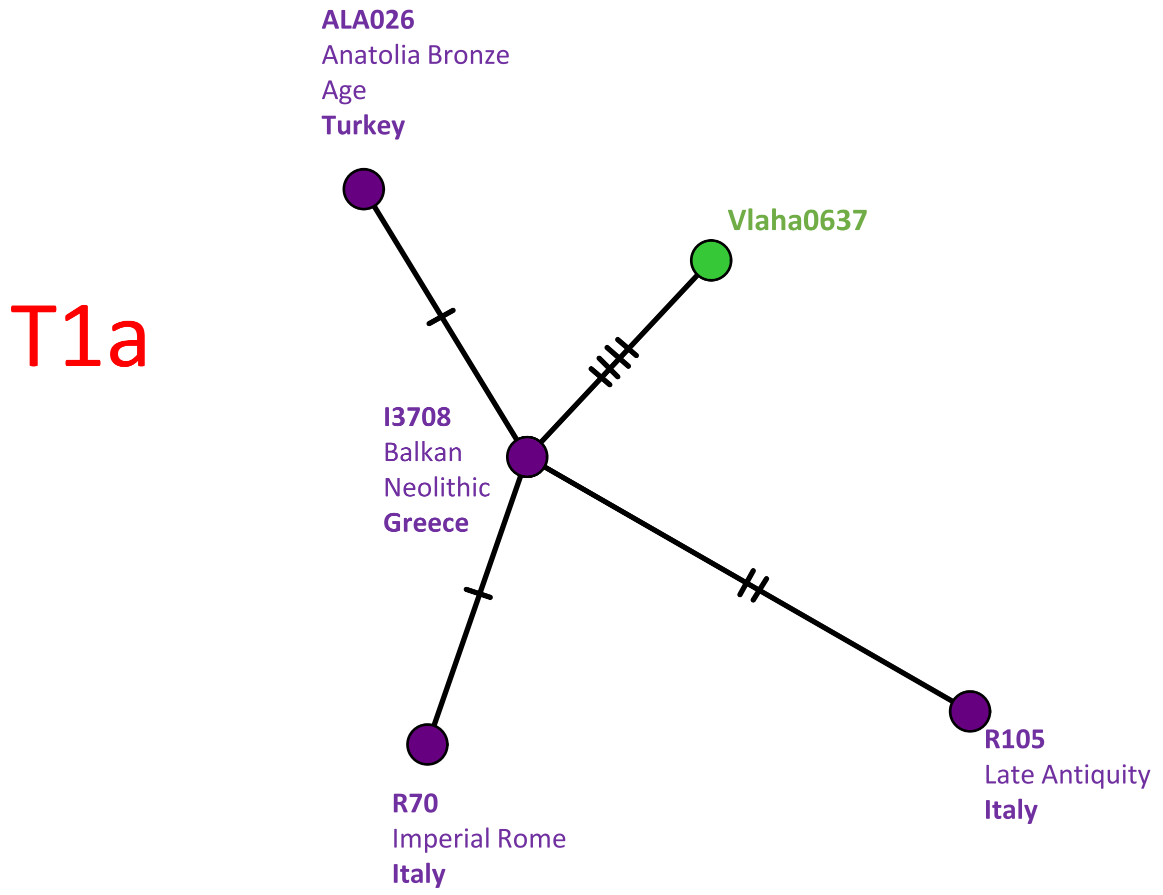
T1a
ALA026
Anatolia Bronze
Age
Turkey
Vlaha0637
I3708
Balkan
Neolithic
Greece
R70
Imperial Rome
Italy
R105
Late Antiquity
Italy

Network 23

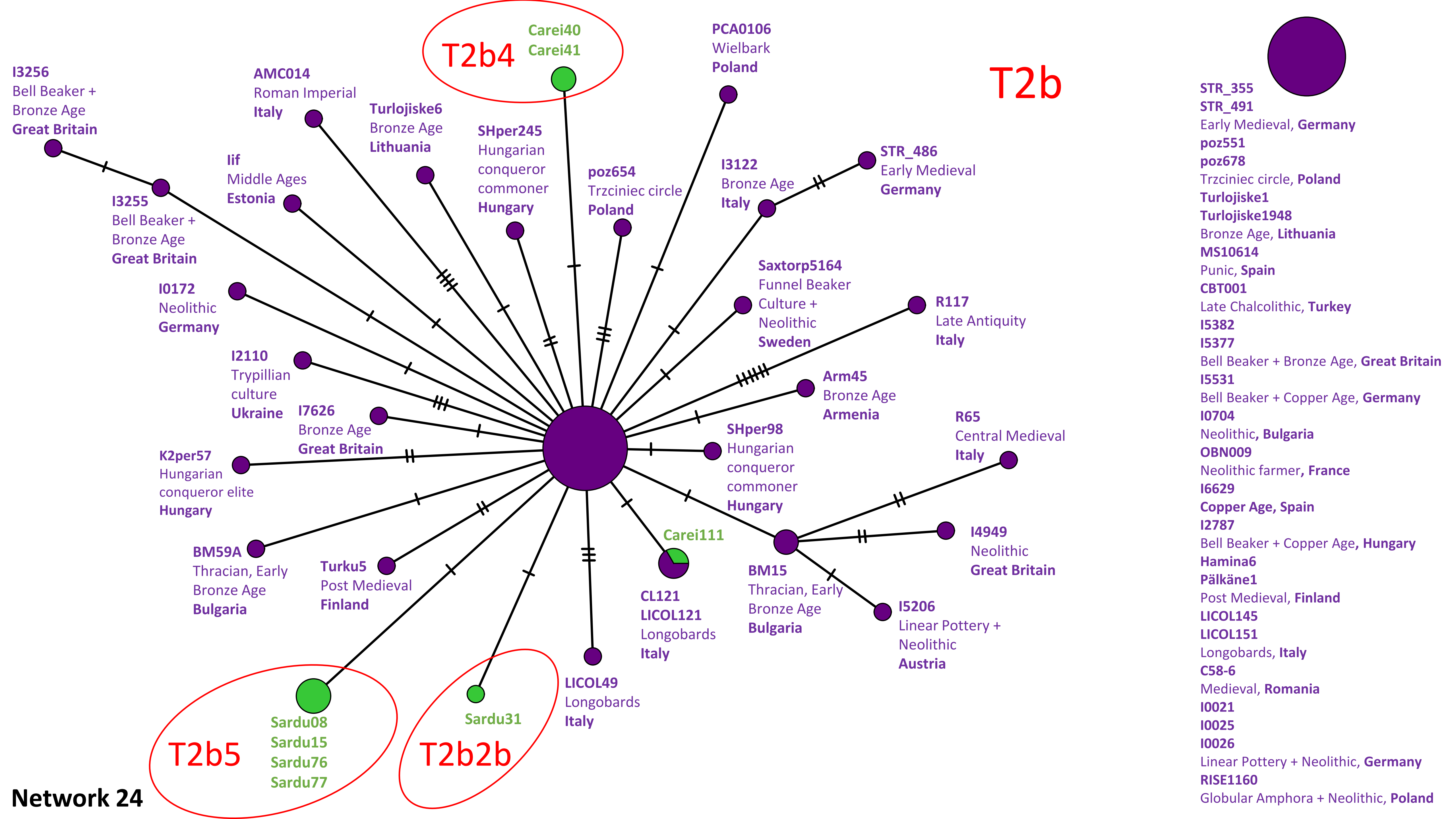

T2b
T2b4
Carei40
Carei41
I3256
Bell Beaker + Bronze Age
Great Britain
AMC014
Roman Imperial
Italy
Turlojiske6
Bronze Age
Lithuania
SHper245
Hungarian conqueror commoner
Hungary
poz654
Trzciniec circle
Poland
PCA0106
Wielbark
Poland
I3122
Bronze Age
Italy
STR_486
Early Medieval
Germany
I3255
Bell Beaker + Bronze Age
Great Britain
lif
Middle Ages
Estonia
I0172
Neolithic
Germany
Saxtorp5164
Funnel Beaker Culture + Neolithic
Sweden
R117
Late Antiquity
Italy
I2110
Trypillian culture
Ukraine
I7626
Bronze Age
Great Britain
Arm45
Bronze Age
Armenia
K2per57
Hungarian conqueror elite
Hungary
SHper98
Hungarian conqueror commoner
Hungary
R65
Central Medieval
Italy
BM59A
Thracian, Early Bronze Age
Bulgaria
Turku5
Post Medieval
Finland
Carei111
CL121
LICOL121
Longobards
Italy
BM15
Thracian, Early Bronze Age
Bulgaria
I4949
Neolithic
Great Britain
I5206
Linear Pottery + Neolithic
Austria
LICOL49
Longobards
Italy
Sardu08
Sardu15
Sardu76
Sardu77
T2b5
Sardu31
T2b2b
STR_355
STR_491
Early Medieval, Germany
poz551
poz678
Trzciniec circle, Poland
Turlojiske1
Turlojiske1948
Bronze Age, Lithuania
MS10614
Punic, Spain
CBT001
Late Chalcolithic, Turkey
I5382
I5377
Bell Beaker + Bronze Age, Great Britain
I5531
Bell Beaker + Copper Age, Germany
I0704
Neolithic, Bulgaria
OBN009
Neolithic farmer, France
I6629
Copper Age, Spain
I2787
Bell Beaker + Copper Age, Hungary
Hamina6
Pälkäne1
Post Medieval, Finland
LICOL145
LICOL151
Longobards, Italy
C58-6
Medieval, Romania
I0021
I0025
I0026
Linear Pottery + Neolithic, Germany
RISE1160
Globular Amphora + Neolithic, Poland

Network 24

T2f1a1

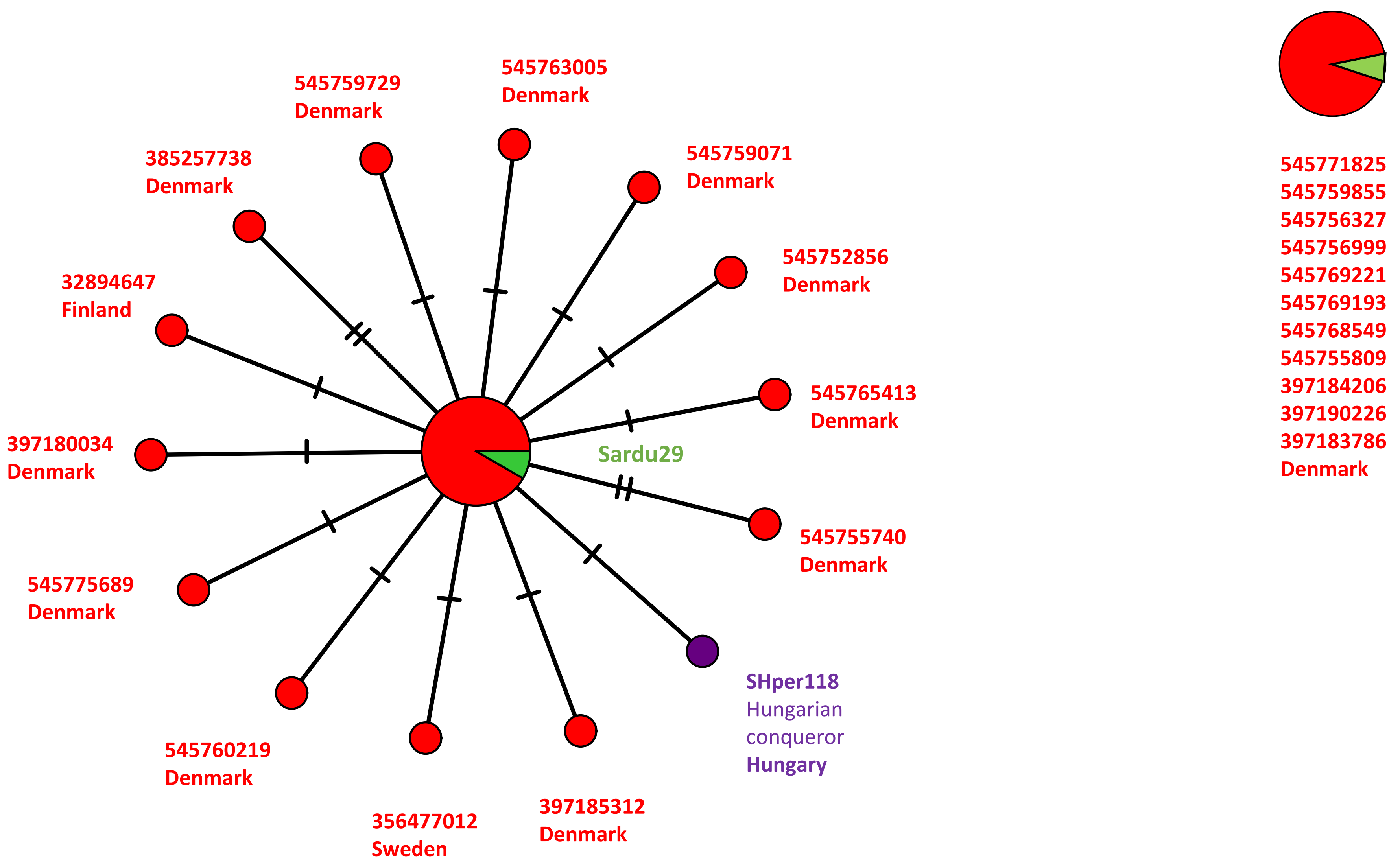

Network 25

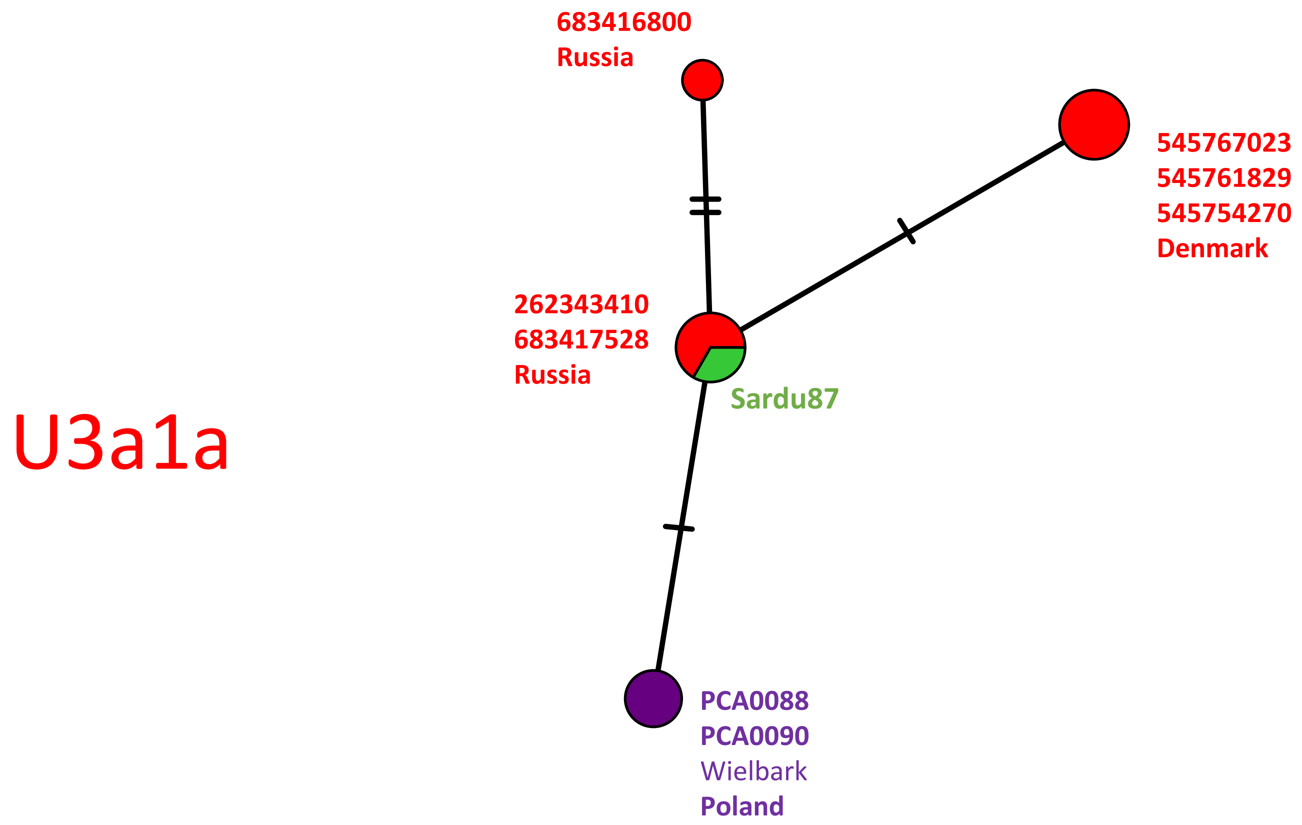
683416800
Russia
545767023
545761829
545754270
Denmark
262343410
683417528
Russia
Sardu87
U3a1a
PCA0088
PCA0090
Wielbark
Poland

Network 26

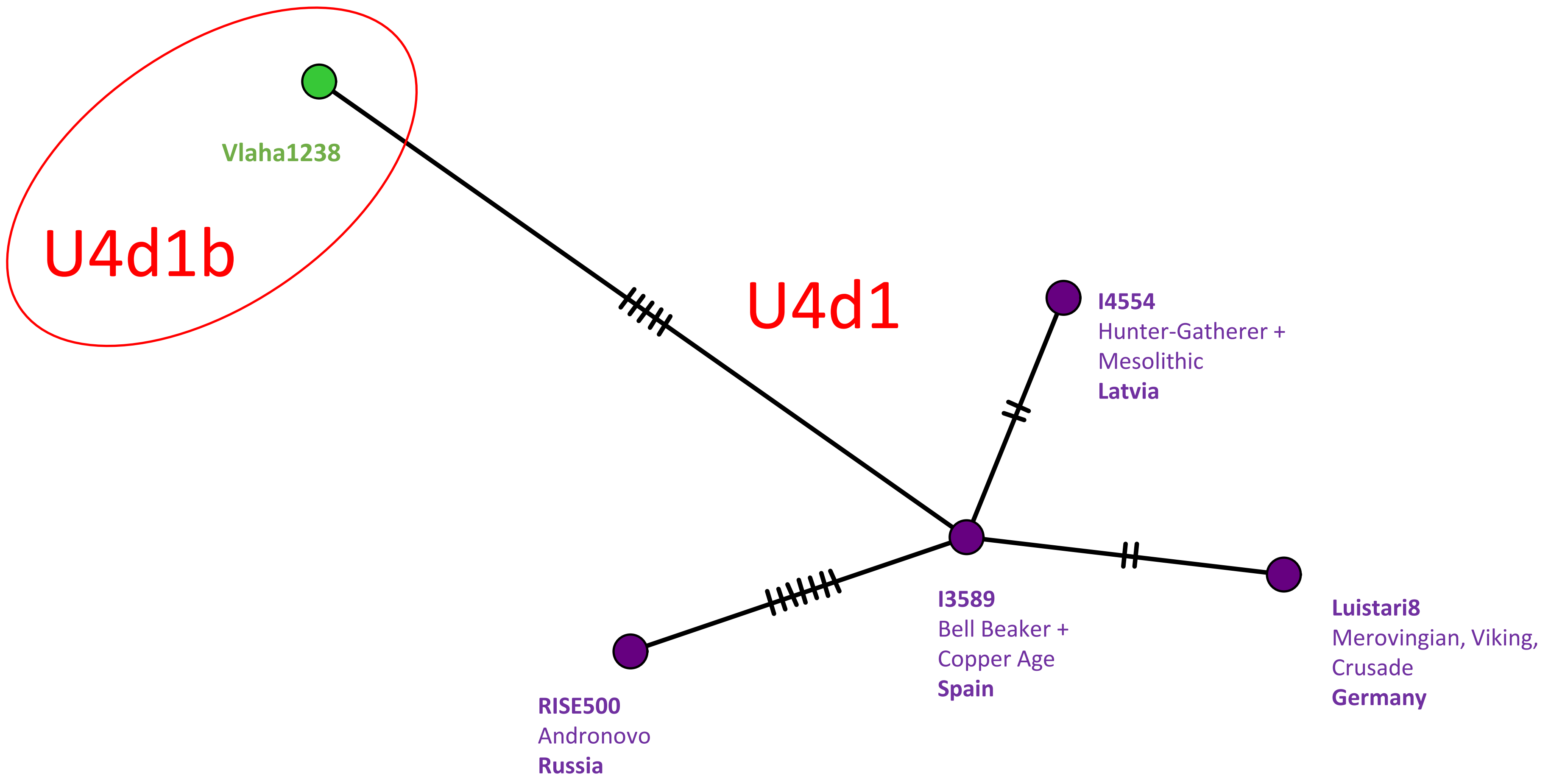
Vlaha1238
U4d1b
U4d1
I4554
Hunter-Gatherer +
Mesolithic
Latvia
I3589
Bell Beaker +
Copper Age
Spain
Luistari8
Merovingian, Viking,
Crusade
Germany
RISE500
Andronovo
Russia

Network 27

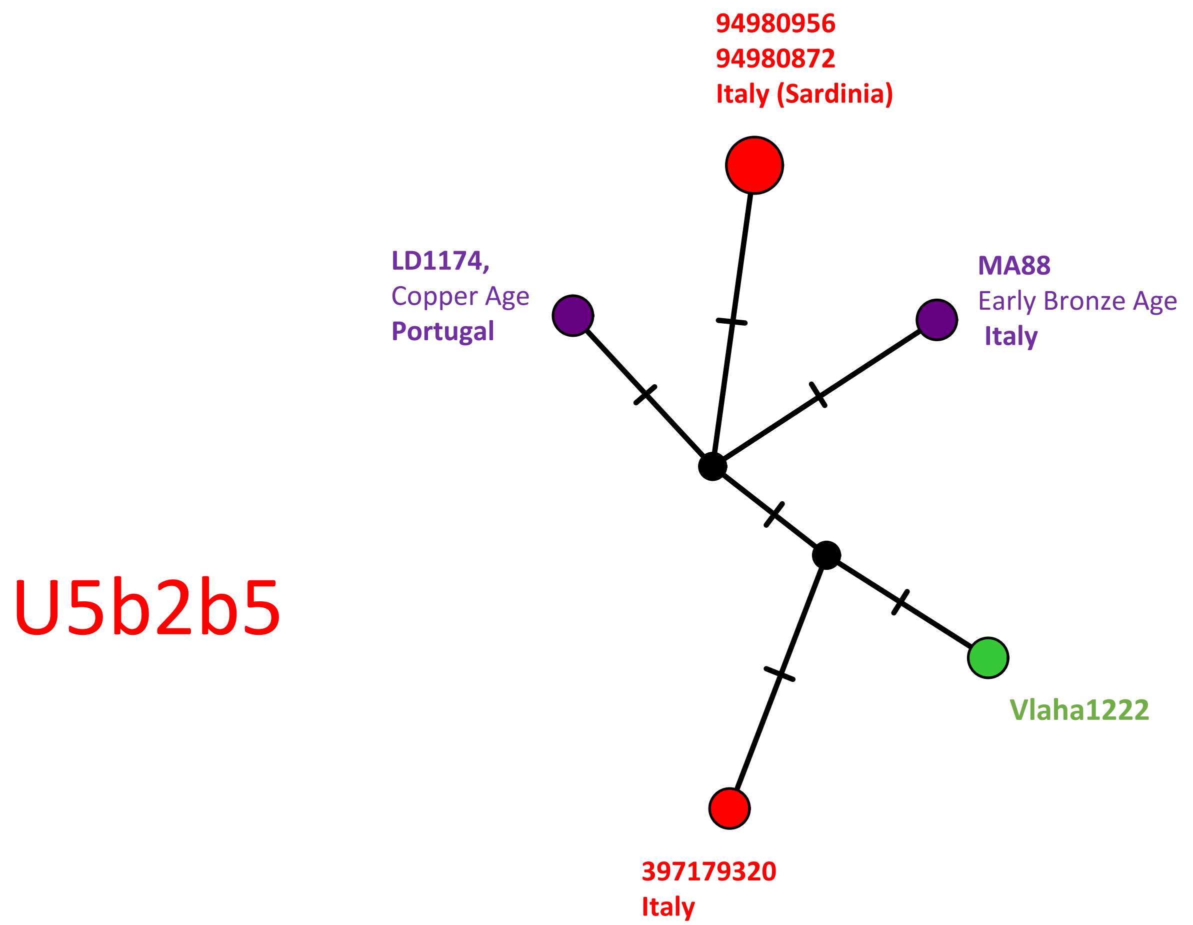
94980956
94980872
Italy (Sardinia)
LD1174,
Copper Age
Portugal
MA88
Early Bronze Age
Italy
U5b2b5
Vlaha1222
397179320
Italy

Network 28

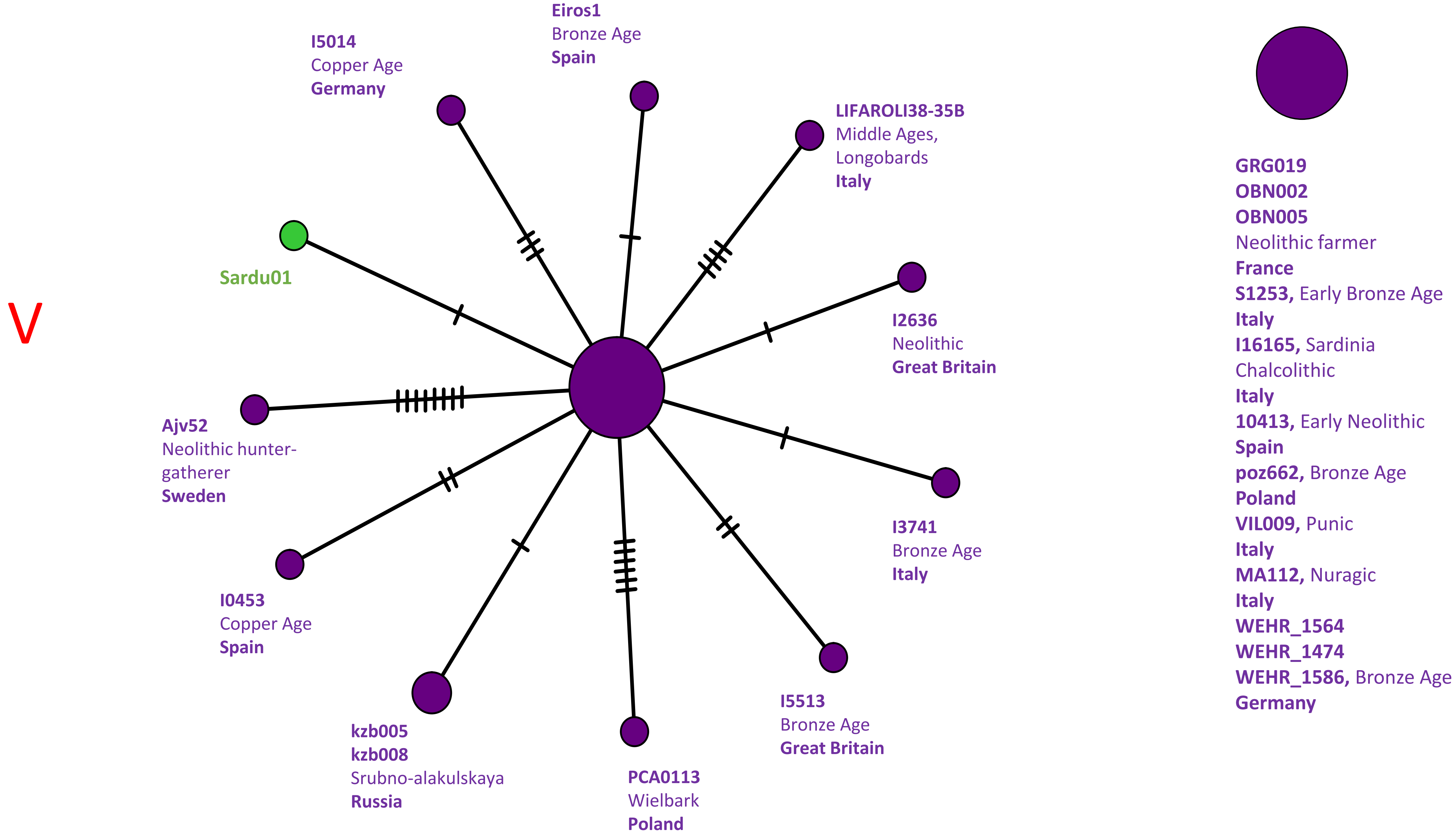
V
I5014
Copper Age
Germany
Eiros1
Bronze Age
Spain
LIFAROLI38-35B
Middle Ages,
Longobards
Italy
Sardu01
I2636
Neolithic
Great Britain
Ajv52
Neolithic hunter-gatherer
Sweden
I3741
Bronze Age
Italy
I0453
Copper Age
Spain
kzb005
kzb008
Srubno-alakulskaya
Russia
PCA0113
Wielbark
Poland
I5513
Bronze Age
Great Britain
GRG019
OBN002
OBN005
Neolithic farmer
France
S1253, Early Bronze Age
Italy
I16165, Sardinia
Chalcolithic
Italy
10413, Early Neolithic
Spain
poz662, Bronze Age
Poland
VIL009, Punic
Italy
MA112, Nuragic
Italy
WEHR_1564
WEHR_1474
WEHR_1586, Bronze Age
Germany

Network 29

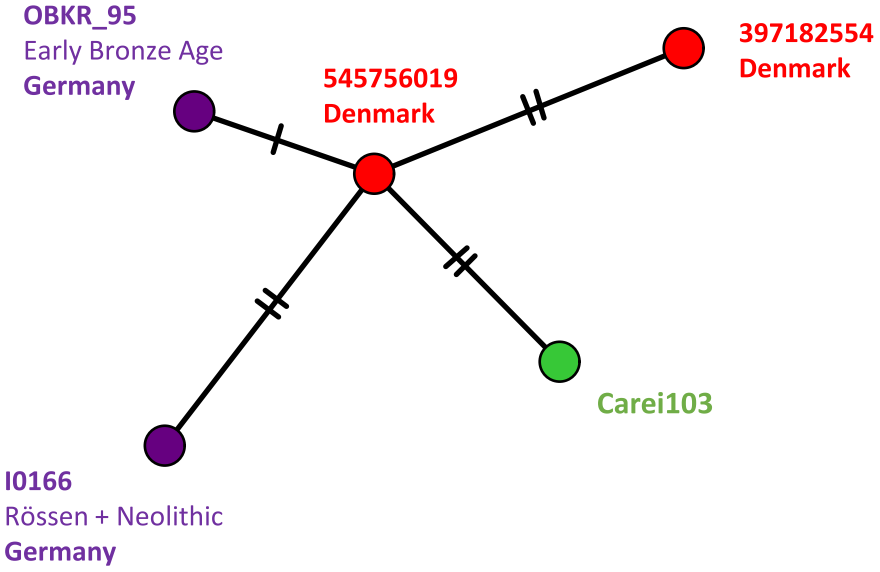

V1a

Network 30
